# Genomic Prediction and the Practical Breeding of 12 Quantitative-Inherited Traits in Cucumber (*Cucumis sativus* L.)

**DOI:** 10.3389/fpls.2021.729328

**Published:** 2021-08-24

**Authors:** Ce Liu, Xiaoxiao Liu, Yike Han, Xi'ao Wang, Yuanyuan Ding, Huanwen Meng, Zhihui Cheng

**Affiliations:** ^1^College of Horticulture, Northwest A&F University, Yangling, China; ^2^State Key Laboratory of Vegetable Germplasm Innovation, Tianjin Key Laboratory of Vegetable Breeding Enterprise, Cucumber Research Institute, Tianjin Academy of Agricultural Sciences, Tianjin, China

**Keywords:** cucumber breeding, genomic prediction, GBLUP, bayesian ridge regression, model validation

## Abstract

Genomic prediction is an effective way for predicting complex traits, and it is becoming more essential in horticultural crop breeding. In this study, we applied genomic prediction in the breeding of cucumber plants. Eighty-one cucumber inbred lines were genotyped and 16,662 markers were identified to represent the genetic background of cucumber. Two populations, namely, diallel cross population and North Carolina II population, having 268 combinations in total were constructed from 81 inbred lines. Twelve cucumber commercial traits of these two populations in autumn 2018, spring 2019, and spring 2020 were collected for model training. General combining ability (GCA) models under five-fold cross-validation and cross-population validation were applied to model validation. Finally, the GCA performance of 81 inbred lines was estimated. Our results showed that the predictive ability for 12 traits ranged from 0.38 to 0.95 under the cross-validation strategy and ranged from −0.38 to 0.88 under the cross-population strategy. Besides, GCA models containing non-additive effects had significantly better performance than the pure additive GCA model for most of the investigated traits. Furthermore, there were a relatively higher proportion of additive-by-additive genetic variance components estimated by the full GCA model, especially for lower heritability traits, but the proportion of dominant genetic variance components was relatively small and stable. Our findings concluded that a genomic prediction protocol based on the GCA model theoretical framework can be applied to cucumber breeding, and it can also provide a reference for the single-cross breeding system of other crops.

## Introduction

Genomic prediction predicts the breeding value of potential hybrids using high-density molecular markers or genetic relationship matrices (Meuwissen et al., 2001; Jannink et al., 2010; Crossa et al., 2017) and is more effective in selecting potential hybrids. The genomic prediction method has been widely used in crop breeding, such as maize (Riedelsheimer et al., 2012), rice (Xu et al., 2016), and potato (Sverrisdottir et al., 2018). However, relatively fewer studies in horticultural crops are reported, such as in pea (Tayeh et al., 2015), tomato (Duangjit et al., 2016), and strawberry (Gezan et al., 2017), while there are still no related reports in cucumber.

Cucumber (*Cucumis sativus* L.) is an important vegetable crop, with a worldwide production of more than 87 million tons in 2019 (http://www.fao.org/faostat/en/?#data/QC). Nowadays, using the traditional breeding methods, diverse ecotypes of cucumber have been developed to satisfy different consumption markets (Weng et al., 2015). Many important horticultural traits, such as size/shape of fruits and flowering time, which are quantitatively inherited and their underlying quantitative trait locus and related genes have been recently reported and identified (Pan et al., 2020). Mostly, the quantitative traits of cucumber are their economic traits that have a polygenic structure and are highly influenced by environmental factors. However, selecting potential hybrids by using the marker-assistant selection method has its limitations under such circumstances. Fortunately, genomic prediction models can capture complex genetic variances using genomic-coverage molecular markers to achieve more accuracy in the selection process. In cucumber, the genomic prediction has also become more practical with the release of the full cucumber genome (Huang et al., 2009; Yang et al., 2012) and the decreasing cost of genome sequencing.

In genomic prediction practices, fitting high-density markers as fixed effects will cause over-fitting issues in a linear model (due to *n* << *p*, where *n* signifies observations and *p* signifies predictors). Therefore, alternatively, they can be fitted as a random effect such as ridge-regression best linear unbiased prediction (BLUP) (Ogutu et al., 2012). To improve the normal distribution assumption of the random effect in ridge-regression BLUP for some traits with more simple inheritance, the Bayesian models may assign different priors to the marker effects allowing for stronger shrinkage or a mixture of null- and major-effect markers (Karkkainen and Sillanpaa, 2012; Perez and de los Campos, 2014). In addition, genetic relationship matrices are used as predictors in the genomic-BLUP (GBLUP) model (Nishio and Satoh, 2014), which can estimate the genetic variance components more conveniently under the Bayesian theoretical framework, and it is equivalent to the ridge-regression BLUP model (Endelman, 2011).

In a single-cross breeding system, F_1_ hybrids produced by the inbred lines are breeding goals, where their performances are divided into general combining ability (GCA) and special combining ability (SCA) to explain the genetic effects from parental independence and its interaction (de Santana et al., 2017). In previous studies, GCA and SCA have been used in cucumber breeding (Golabadi et al., 2015; Moradipour et al., 2017; Ene et al., 2019), which indicates that both GCA and SCA are good indicators for evaluating either the inbred lines or hybrid lines. Furthermore, the accurate estimation of combining ability relies on the orthogonal *a priori* hypothesis of variance components, and the GCA model has been developed for the single-cross breeding system (González-Diéguez et al., 2021). In the GCA model, the genetic relationship matrices of additive, dominance, additive-by-additive, and residual genetic effects are defined orthogonally. The GCA model has shown its potential in a maize single-cross breeding program (Technow et al., 2014; González-Diéguez et al., 2021). In this study, we investigated whether the GCA model is suitable for the cucumber single-cross breeding system.

In general, the predictive ability of the model is estimated by the cross-validation (Heslot et al., 2012). In some cases, the model performance needs more comprehensive verification. The performance of a model for cross seasons and/or cross populations is necessary to validate since the population size and the times of trial in the field are usually limited in a training group. From another perspective, whether the genetic variance components are similar among different seasons and/or population structures is also essential for the comprehension of trait performance in a breeding system.

To verify the applicability of genomic prediction in cucumber, three GCA models under two model validation strategies in cucumber were performed to solve the following issues: (i) estimation of variance parameters under orthogonal prior hypothesis; (ii) the GCA model performance under cross-validation and cross-population strategies; and (iii) estimation of the GCA performance of 81 inbred lines.

## Materials and Methods

### Construction of Model Training Populations

Seventy-one cucumber inbred lines were collected for genomic prediction. For these collected lines, the inbred lines with “WI ##” were provided by professor Yiqun Weng at the University of Wisconsin-Madison, and most of the other inbred lines were obtained from our own Laboratory. Besides, another 10 inbred lines from Liu et al. (2015) and Qi et al. (2013) were used for the analysis. The origin of some inbred lines refers to Bo et al. (2016), and the detailed information of all 81 inbred lines is listed in Supplementary Table 1.

There were two populations for model training, namely, diallel cross (DC) population and the North Carolina II (NC) population. The DC population included 18 inbred lines, 153 DC F_1_ hybrids, and another 5 F_1_ hybrids developed from 8 cucumber inbred lines with a total of 176 combinations. The NC population included 92 combinations, which were derived by “23,” “3,511” as male parents, and other 49 inbred lines as female parents based on the NC genetic mating design (Xu et al., 2018). The detailed information for two model training populations is listed in Supplementary Table 2.

### Genotypic Data Collection

Seventy-two collected inbred lines were planted in a greenhouse at the Horticultural Farm of Northwest A&F University (HF-NWAF), Yangling, Shaanxi Province, China, (108.0809°E, 34.3006°N) during spring 2018. Young leaves of these inbred lines were collected for DNA extraction by using the cetyl trimethyl ammonium bromide (CTAB) method from the seedlings at a four-leaf stage. Murray and Thompson (1980). The qualified DNA samples were used for sequencing library construction following the paired-end library protocol (Illumina company). The Illumina HiSeq2500 platforms and the paired-end 150 bp sequencing strategy were used for re-sequencing.

To obtain clean reads, raw sequencing data were filtered by removing sequences with adaptors, filtering out the reads with N content over 10%, and ignoring the reads that had over 50% of low-quality bases. Regarding reads alignment and single nucleotide polymorphism (SNP) calling, 81 inbred lines were sequenced and analyzed altogether. First, the clean reads of 81 inbred lines were aligned onto the draft genome of cucumber “9930” V2.0 (https://www.ncbi.nlm.nih.gov/genome/1639?genome_assembly_id=228904), using BWA software (Li and Durbin, 2010) (parameter: mem -t 4 -k 32 -M). Second, the SNP calling process was performed by using SAMtools software (Li et al., 2009) with the “mpileup” function. Missing and heterozygous SNP loci were imputed by major homozygous SNP genotype, and the SNP locus with minor allele frequency <0.05 was removed. Later, the SNPs located on coding sequences were annotated by using ANNOVAR software (Wang et al., 2010). Finally, 16,662 non-synonymous SNPs were collected as genotypic data for the construction of genetic relationship matrices. The origin SNP information of 81 inbred lines is given in Supplementary File 1.

### Phenotypic Data Collection

Three field trials of the DC population were carried out during autumn 2018 (2018A), spring 2019 (2019S), and spring 2020 (2020S). The planting dates of these three seasons are July 27, 2018, March 14, 2019, and March 6, 2020, respectively. One field trial of the NC population was carried out during 2020S. For each field trial, combinations in the DC population had two blocks (separated glasshouse as a block) while four (two blocks in a single glasshouse) in the NC population. A total of 10 plants were sowed for each combination in a block, and the detailed experimental design was visualized in Supplementary Figure 1.

A total of 12 traits were collected in both DC and NC populations, namely, commercial fruit yield (cFY, kg), commercial fruit number (cFN), female flower time (FFT, days), commercial fruit weight (cFW, g), commercial fruit length (cFL, mm), commercial fruit diameter (cFD, mm), commercial fruit neck length (cFNL, mm), commercial fruit flesh thickness (cFTH, mm), commercial seed cavity radius (cSCR, mm), commercial fruit spine density (cFSD, n·cm^−2^), female flower node ratio (FFNR), and the first female flower node (FFFN) traits. The naming standard of these traits follows the rules in the study by Wang et al. (2020).

The commercial fruits of all combinations in three seasons were collected and measured according to the market standard of fresh cucumbers (Golabadi et al., 2015). The phenotypic data collection methods were described as follows:

For cFY and cFN, the total cFY and cFN were continuously recorded up to 30 harvesting days. FFT was recorded from sowing until days to 50% of female flowering. Moreover, each block was a measurement unit for cFY, cFN, and FFT. FFNR was recorded by calculating a ratio of female flower nodes within the first 20 nodes and FFFN from the FFFN. A total of six plants were used for measuring FFNR and FFFN, while a total of six commercial fruits were collected for the measurement of cFW, cFL, cFD, cFNL, cFTH, cSCR, and cFSD, respectively. cFSD trait was recorded by selecting six random fruits, and their spine numbers were converted to spine density (n·cm^−2^). All these 12 traits were recorded in 2019S and 2020S; however, the FFNR and FFFN traits were not available in 2018A. The information of origin phenotypic data are listed in Supplementary File 2.

Before model training, for most traits in each season, including cFW, cFL, cFD, cFNL, cFTH, cSCR, cFSD, FFNR, and FFFN, the phenotypic data were corrected using the following mixed linear model:

(1) $$\begin{array}{l} y_{ijk}=g_{i}+block_{j}+Rep_{k}+\varepsilon_{ijk} \end{array}$$

where *y*_*ijk*_ is the origin phenotypic value, *g*_*i*_ is the best linear unbiased estimates (BLUEs) of the *i*th hybrids, *block*_*j*_ is the *j*th block, *Rep*_*k*_ is the *k*th replicates, and ε_*ijk*_ is the residual item. For cFY, cFN, and FFT in each season, the phenotypic data were corrected using a simplified model: *y*_*ijk*_ = *g*_*i*_ + *block*_*j*_ + ε_*ijk*_, where the replicate item was ignored.

### Model Type and Model Validation Strategy

For a single-cross breeding system, the decomposition of combining ability of F_1_ hybrids is the basic theoretical framework of genomic prediction, and the basic formula is:

(2) $$\begin{array}{l} y_{ij}=\mu +GCA_{i}+GCA_{j}+SCA_{ij}+\varepsilon_{ij} \end{array}$$

where *y*_*ij*_ is the BLUEs of F_1_ combinations (female parent *i* × male parent *j*), μ is the overall mean of the group, *GCA*_*i*_ and *GCA*_*j*_ are the GCA of female parent (*i*) and male parent (*j*), respectively. *SCA*_*ij*_ is the SCA of the F_1_ hybrids (female parent *i* × male parent *j*), *and ε*_*ij*_ is the residual item.

In this study, the GCA models developed by González-Diéguez et al. (2021) were used for model training. In the full GCA model, additive, dominance, additive-by-additive epistatic, and residual genetic effects were all considered. In addition, GCA components were considered by the following formula:

(3) $$\begin{array}{l} GCA=T_{1}g_{A}+T_{2}g_{A}+T_{1}g_{AA}+T_{2}g_{AA}+T_{1}r+T_{2}r \end{array}$$

where *GCA* is the GCA effect vector of inbred lines, *g*_*A*_ is the additive effect vector of inbred lines, *g*_*AA*_ is the additive-by-additive (intrapopulation epistatic) effect vector of inbred lines, and *T*_1_ and *T*_2_ are incidence matrices assigning hybrids to female parents and male parents, respectively. The additive effects in inbred line population are assumed to be distributed as *g*_*A*_ ~ $MVN\left(0,\;G_{A}\sigma_{A}^{2}\right)$, and the *G*_*A*_ matrix was calculated based on the following formula: $G_{A}=\frac{ZZ^{\prime }}{\overset{n}{\underset{i}{\sum }}p_{i}q_{i}}$, where *p*_*i*_ and *q*_*i*_ are allele frequencies in *i*th SNP loci and *n* is the SNP number. $Z=M-1_{n}p\prime$, where *M* is the *m* × *n* SNP matrix of inbred lines, and *m* is the inbred line number. {0, 1} are basic elements in *M*, where 0 means “aa” and 1 means “AA.” The intrapopulation additive-by-additive effects in inbred line population are assumed as *g*_*AA*_ **~** $MVN\left(0,\;G_{AA}\sigma_{AA}^{2}\right)$. *r* is the residual genetic effect (Endelman et al., 2018), which is assumed to be distributed as $r\sim MVN\left(0,\;I\sigma_{r}^{2}\right)$, and *r* = *g*_*AAA*_ + *g*_*AAAA*_ + ….

For the SCA component, the dominance effect was considered, and the formula is

(4) $$\begin{array}{l} SCA=g_{D} \end{array}$$

where *SCA* is the SCA effect vector of hybrids, *g*_*D*_ is the dominance effect of hybrids, which is assumed as *g*_*D*_ ~ $MVN\left(0,\;G_{D}\sigma_{D}^{2}\right)$. *G*_*AA*_ and *G*_*D*_ genetic relationship matrices are constructed followed by González-Diéguez et al. (2021).

Thus, the full GCA model is the GCA additive–dominance–epistasis (A–D–E) model, and the formula is

(5) $$\begin{array}{l} y=1_{n}\mu +T_{1}g_{A}+T_{2}g_{A}+{g_{D}+T}_{1}g_{AA}+T_{2}g_{AA}+T_{1}r+T_{2}r+\varepsilon \end{array}$$

where *y* is the BLUE vector of F_1_ combinations, ε is the residual item, and $\varepsilon \sim MVN\left(0,\;I\sigma_{e}^{2}\right)$. Other items in formula (5) have been described earlier.

Besides, the other two simplified models were also used for the analysis as follows:

(6) $$\begin{array}{l} y=1_{n}\mu +T_{1}g_{A}+T_{2}g_{A}+g_{D}+T_{1}r+T_{2}r+\varepsilon \end{array}$$

(7) $$\begin{array}{l} y=1_{n}\mu +T_{1}g_{A}+T_{2}g_{A}+T_{1}r+T_{2}r+\varepsilon \end{array}$$

Formula (6) is the GCA additive–dominance (A–D) model, which contains additive, dominance, and residual genetic effects. Formula (7) is the GCA additive (A) model, which contains additive and residual genetic effects. In addition, three GCA models without residual genetic effects were also performed.

All the three GCA models were performed by using the Bayesian ridge regression framework, and variance components including $\sigma_{A}^{2}$, $\sigma_{D}^{2}$, $\sigma_{AA}^{2}$, $\sigma_{r}^{2}$, and $\sigma_{e}^{2}$ were estimated by using the Bayesian ridge regression model. Broad-sense heritability (*H*^2^) was calculated by the proportion of genetic variance in the total variance. To show the genetic relationship among 81 inbred lines, *G*_*A*_ matrix was visualized using a heat map. Finally, the GCA of 81 inbred lines in all three seasons was estimated using the full GCA model.

**Figure 1:**
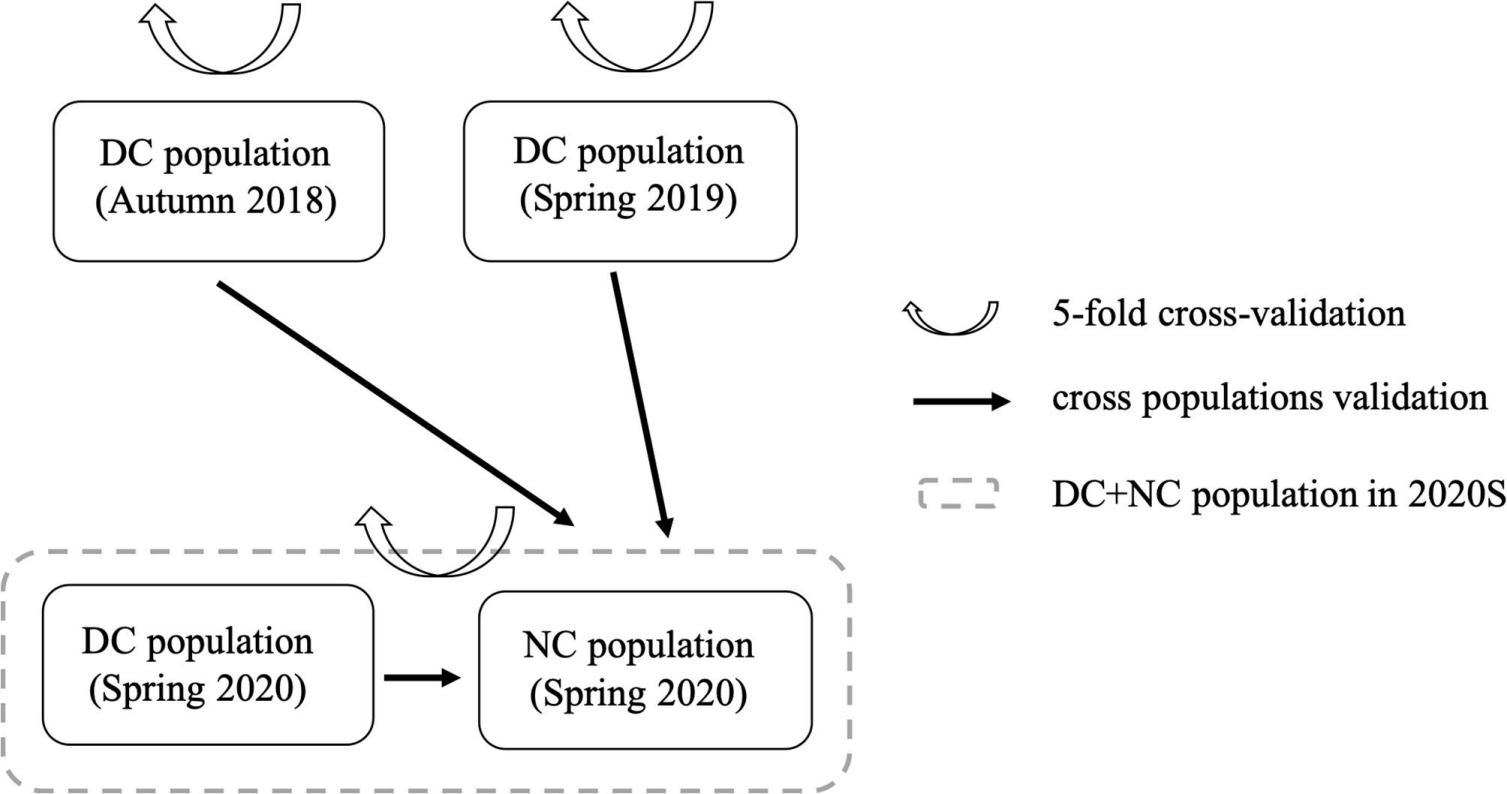
Two model validation strategies for analysis. DC, diallel cross population; NC, NCII population.

Two model validation strategies, namely, five-fold cross-validation and cross-population validation, were used to check the predictive ability of the model. These two strategies are shown in Figure 1.

For the five-fold cross-validation strategy, DC population in 2018A, DC population in 2019S, and (DC + NC) populations in 2020S were training groups, respectively. For the cross-population validation strategy, the DC population in 2018A, 2019S, and 2020S were the training groups, and the NCII population in 2020S was the validation group. Regarding each model validation strategy, the Pearson's correlation relationship between the phenotypic BLUEs and the genomic estimated breeding values (GEBVs) was calculated as model predictive ability, and each model validation process was repeated 20 times to obtain robust results.

### Statistical Analysis

In this study, most of the analyses were performed by using R (3.6.1 version) software (R Development Core Team, 2013). The correlations among the BLUEs of traits in three seasons were visualized by using “*ggpairs*” (https://ggobi.github.io/ggally/reference/ggpairs.html), and the heat map of the *G*_*A*_ matrix was visualized by “*pheatmap*” package using R software (https://CRAN.R-project.org/package=pheatmap). The principal component analysis of DC and NC populations was performed based on the 16,662 non-synonymous SNP information of these two populations using “*prcomp*” R function.

Regarding the GCA models validation process, the “*BGLR*” function in “*BGLR*” package in R software was used for the analysis (Perez and de los Campos, 2014). For the parameter setting of “*BGLR*” function, the *nIter* parameter was 30,000, the *burnIn* parameter was 10,000, and the *thin* parameter was 5 (default value). The trace plot of each variance parameter was visualized to check its convergence. The R script including the function for the model validation process is presented in Supplementary File 3.

## Results

### Overview of Genetic Relationship of Inbred Lines and Phenotypic Data

**Figure 2:**
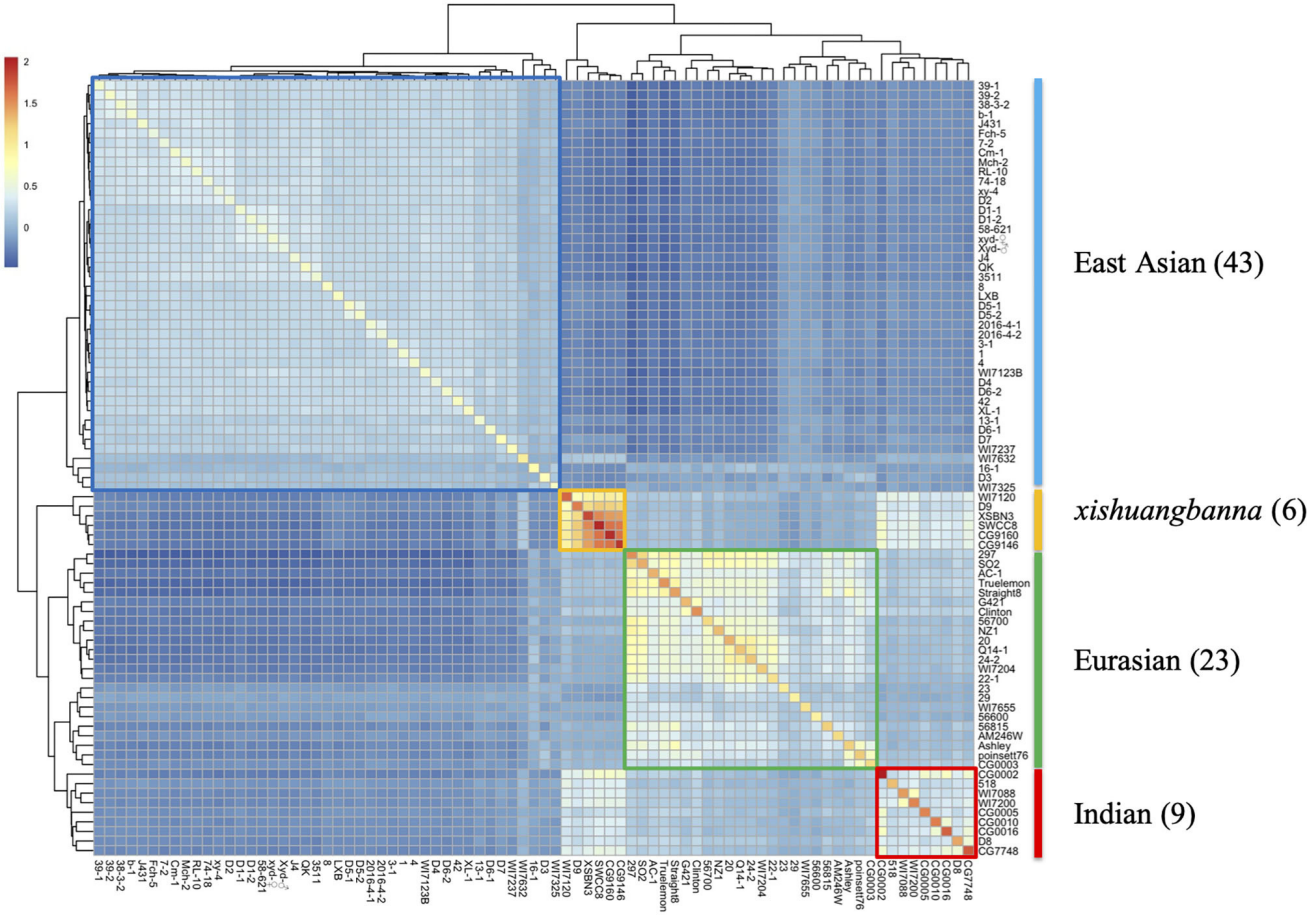
The heat map of the *G*_*A*_ matrix for the dataset with 81 cucumber inbred lines genotyped with 16,662 SNP markers. All 81 cucumber lines are clustered into four groups, namely, East Asia, Eurasian, Indian, and *xishuangbanna* groups, respectively.

In this study, a total of 81 cucumber inbred lines were collected to represent the main genetic background of cucumber. The heat map of *G*_*A*_ matrix (Figure 2) showed that all the collected cucumber inbred lines were divided into four genetic groups, namely, East Asian group (43 inbred lines), Eurasian group (23 inbred lines), Indian group (9 inbred lines), and *xishuangbanna* group (6 inbred lines). Two populations, namely, DC and NC, were constructed for genomic prediction (Supplementary Figure 2A). The principal component analysis of two populations (Supplementary Figure 2B) showed that the DC population had a similar but broader genetic background compared with the NC population.

Two main types of traits in DC and NC populations were collected, including fruit yield-related and commercial-related traits. Fruit yield-related traits included cFY, cFN, FFT, FFNR, and FFFN traits; fruit commercial-related traits included cFW, cFL, cFD, cFNL, cFTH, cSCR, and cFSD traits.

**Figure 3:**
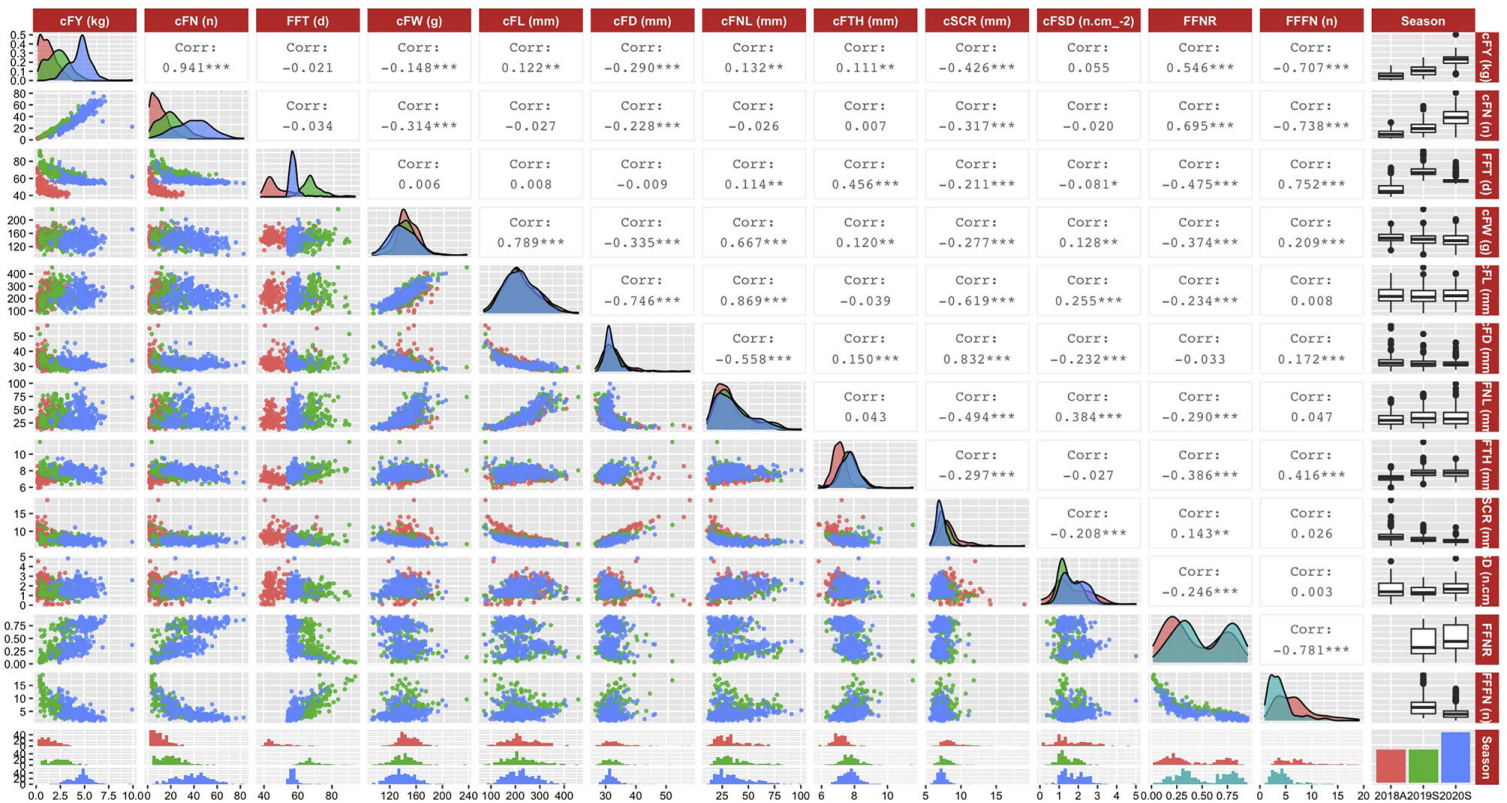
Distribution and correlation relationship among best linear unbiased estimates of traits in three seasons. A total of 12 traits information in three seasons is visualized. The upper right part is the Pearson's correlation coefficient among traits (***: *p* < 0.001; **: *p* < 0.01; and *: *p* < 0.05); the diagonal position is the density distribution of traits in three seasons; and the lower left part is the scatter plot among traits in different seasons. Red color, traits in autumn 2018; green color, traits in spring 2019; blue color, traits in spring 2020.

The distribution and correlation relationship among the BLUEs of traits of DC population (Figure 3) showed that in all the three seasons, cFY paired cFN (*r* = 0.941^***^), cFW paired cFL (*r* = 0.789^***^), cFL paired cFNL (*r* = 0.869^***^), and cSCR paired cFD (*r* = 0.832^***^) had strong positive relationships; cFL paired cFD (*r* = −0.746^***^), cFL paired cSCR (*r* = −0.619^***^), FFNR paired FFFN (*r* = −0.781^***^), and cFN paired FFFN (*r* = −0.781^***^) had strong negative relationships. In addition, the density distributions of cFY, cFN, and FFT traits in all three seasons were different, while most of the fruit commercial-related traits have similar distributions in all three seasons, especially for cFL and cFNL traits. It is noted that the density distribution of cFTH trait in 2018A has a significant deviation compared with those in 2019S and 2020S.

### Model Predictive Ability Under Five-Fold Cross-Validation Strategy

For the five-fold cross-validation strategy, the model predictive ability was estimated by the training group itself. Table 1 shows that for model comparison in 2018A, the GCA (A) model had the best performance for FFT, cFL, cFD, and cFTH, and the GCA (A–D) model had the best performance for cFY, cFN, and cFNL, while the GCA (A–D–E) model had the best performance for cFW, cSCR, cFL, and cFSD. For model comparison in 2019S, the GCA (A) model performed the best for only cFTH and cSCR traits, and the GCA (A–D) model performed the best for cFW, cFD, cFSD, and FFFN traits, while the GCA (A–D–E) model performed the best for the other six traits. For model comparison in 2020S, only the cSCR and cFSD with the GCA (A) model had the best performance, and FFT, cFW, cFL, cFD, FFNR, and FFFN with the GCA (A–D) model had the best performance, while cFY, cFN, cFNL, and cFTH with the GCA (A–D–E) model had the best performance.

**Table 1:** Predictive ability of GCA models under the five-fold cross-validation strategy.

**Season**	**Trait**	**GCA model**		
		**GCA (A)**	**GCA (A–D)**	**GCA (A–D–E)**
2018A	cFY	0.683(0.001) c	0.701(0.001) a	0.697(0.001) b
	cFN	0.654(0.001) c	0.691(0.001) a	0.681(0.001) b
	FFT	0.830(0.001) a	0.799(0.001) b	0.799(0.001) b
	cFW	0.533(0.001) b	0.511(0.001) c	0.592(0.001) a
	cFL	0.912(0.000) a	0.908(0.000) b	0.899(0.000) c
	cFD	0.840(0.000) a	0.825(0.001) c	0.828(0.001) b
	cFNL	0.885(0.000) c	0.901(0.000) a	0.895(0.000) b
	cFTH	0.439(0.001) a	0.426(0.001) b	0.381(0.002) c
	cSCR	0.826(0.000) c	0.831(0.001) b	0.831(0.001) a
	cFSD	0.803(0.001) b	0.802(0.001) c	0.811(0.001) a
2019S	cFY	0.715(0.001) c	0.782(0.001) b	0.796(0.001) a
	cFN	0.788(0.001) c	0.814(0.001) b	0.823(0.001) a
	FFT	0.729(0.001) c	0.768(0.001) b	0.783(0.002) a
	cFW	0.846(0.000) c	0.857(0.000) a	0.848(0.000) b
	cFL	0.948(0.000) c	0.955(0.000) b	0.957(0.000) a
	cFD	0.882(0.000) b	0.886(0.000) a	0.880(0.001) c
	cFNL	0.913(0.000) c	0.920(0.001) b	0.924(0.000) a
	cFTH	0.679(0.001) a	0.663(0.001) c	0.672(0.001) b
	cSCR	0.860(0.000) a	0.854(0.000) b	0.853(0.000) c
	cFSD	0.866(0.000) c	0.882(0.000) a	0.873(0.001) b
	FFNR	0.866(0.000) c	0.897(0.000) b	0.898(0.001) a
	FFFN	0.811(0.001) b	0.836(0.001) a	0.785(0.001) c
2020S	cFY	0.725(0.001) c	0.738(0.001) b	0.779(0.000) a
	cFN	0.845(0.001) c	0.886(0.001) b	0.900(0.000) a
	FFT	0.482(0.002) c	0.650(0.002) a	0.620(0.002) b
	cFW	0.840(0.001) b	0.845(0.001) a	0.836(0.000) c
	cFL	0.950(0.000) c	0.955(0.001) a	0.952(0.001) b
	cFD	0.827(0.001) c	0.839(0.001) a	0.832(0.000) b
	cFNL	0.892(0.000) c	0.903(0.001) b	0.913(0.000) a
	cFTH	0.668(0.001) b	0.668(0.001) b	0.680(0.001) a
	cSCR	0.805(0.001) a	0.805(0.001) a	0.798(0.001) b
	cFSD	0.791(0.000) a	0.782(0.001) b	0.744(0.002) c
	FFNR	0.846(0.000) c	0.880(0.000) a	0.875(0.000) b
	FFFN	0.752(0.000) c	0.792(0.001) a	0.791(0.001) b

*Regarding the GCA model, (A) is an additive model, (A–D) is an additive–dominance model, and (A–D–E) is the additive–dominance–epistasis (additive-by-additive) model. The predictive ability is presented as mean (SD). Different letters indicate a significant difference in the predictive abilities of different models for the same trait (p < 0.05, Duncan's new multiple range test)*.

*GCA, general combining ability; cFY, commercial fruit yield; cFN, commercial fruit number; FFT, female flowering time; cFW, commercial fruit weight; cFL, commercial fruit length; cFD, commercial fruit diameter; cFNL, commercial fruit neck length; cFTH, commercial fruit flesh thickness; cSCR, commercial seed cavity radius; cFSD, commercial fruit spine density; FFNR, female flower node ratio; FFFN, first female flower node*.

Besides, GCA models had a better predictive ability for most of the fruit commercial-related traits in three seasons, except for the cFTH. Moreover, the GCA models for cFL had the highest predictive ability (*r* > 0.90 in all three seasons) compared with the other 11 traits, while the GCA model had a relatively worse predictive ability for cFTH in 2018A (0.38–0.44) and FFT in 2020S (0.48–0.62).

### The Estimation of Variance Parameters of GCA Models

The posterior genetic variance parameters of three GCA models for 12 traits in three seasons were estimated. The information of variance parameters for the cFY trait is listed in Table 2, and the information of variance parameters for the other 11 traits is listed in Supplementary Table 3. For all the 12 traits, with increasing model complexity, a higher proportion of genetic variance components was observed, leading to a higher broad-sense heritability. For example, for the cFY in 2018A, broad-sense heritability showed increment by 11.6% under the GCA (A–D–E) model (0.37) as compared with the GCA (A) model (0.33).

**Table 2:** The estimated posterior of genetic variance components and broad-sense heritability (*H*^2^) under three GCA models for the cFY trait.

**Season**	**Model**	**Variance components**					***H^**2**^***
		$\sigma_{A}^{2}$	$\sigma_{D}^{2}$	$\sigma_{AA}^{2}$	$\sigma_{r}^{2}$	$\sigma_{\varepsilon }^{2}$	
2018A	GCA (A)	0.028 (0.022)			0.122 (0.054)	0.306 (0.034)	0.328
2018A	GCA (A-D)	0.023 (0.021)	0.011 (0.008)		0.111 (0.053)	0.282 (0.033)	0.339
2018A	GCA (A–D–E)	0.013 (0.012)	0.008 (0.005)	0.071 (0.044)	0.071 (0.035)	0.282 (0.033)	0.366
2019S	GCA (A)	0.082 (0.054)			0.243 (0.113)	0.433 (0.049)	0.429
2019S	GCA (A-D)	0.064 (0.044)	0.022 (0.013)		0.219 (0.115)	0.374 (0.044)	0.449
2019S	GCA (A–D–E)	0.033 (0.039)	0.018 (0.011)	0.169 (0.102)	0.136 (0.076)	0.372 (0.043)	0.488
2020S	GCA (A)	0.123 (0.057)			0.275 (0.087)	0.389 (0.042)	0.506
2020S	GCA (A–D)	0.162 (0.073)	0.012 (0.006)		0.275 (0.094)	0.309 (0.034)	0.593
2020S	GCA (A–D–E)	0.035 (0.038)	0.009 (0.004)	0.176 (0.092)	0.225 (0.075)	0.311(0.033)	0.589

*Regarding the GCA model, (A) is an additive model, (A–D) is an additive–dominance model, and (A–D–E) is the additive–dominance–epistasis (additive-by-additive) model*.

$\sigma_{A}^{2}$, $\sigma_{D}^{2},\;\sigma_{AA}^{2}$, *and*$\sigma_{r}^{2}$*are additive, dominance, additive-by-additive, and residual genetic variance components, respectively*. $\sigma_{\varepsilon }^{2}$*is the residual variance*.

*The estimated posterior of genetic variance components is expressed as mean (SD)*.

*GCA, general combining ability; cFY, commercial fruit yield*.

Some traits in different seasons had obvious differences for broad-sense heritability. For example, for cFY in 2018A, the broad-sense heritability under the GCA (A–D–E) model was 0.37, and in 2019S, the broad-sense heritability under the GCA (A–D–E) model was 0.49. Besides, the cFTH in 2018A had low broad-sense heritability (0.19–0.22) compared with the other two seasons (0.34–0.43).

In addition, a relatively high proportion of additive-by-additive (epistatic) genetic variance was captured in the GCA (A–D–E) model for most of the traits. For example, for cFY in 2018A, the additive-by-additive genetic variance accounted for 43.5% of the total genetic variance under the GCA (A–D–E) model and for cFY in 2019S, the ratio was 47.6%. And in 2020S, the ratio was 39.6%. But for the high broad-sense heritability traits, like cFL in 2020S, the ratio of additive-by-additive genetic variance to total genetic variance was only 20.6%. When $\sigma_{AA}^{2}$ existed (GCA (A–D–E) model), the proportion of additive and residual genetic components drops compared with the other GCA models. In contrast, the proportion of dominant genetic variance in total genetic variance during three GCA models is relatively stable.

**Figure 4:**
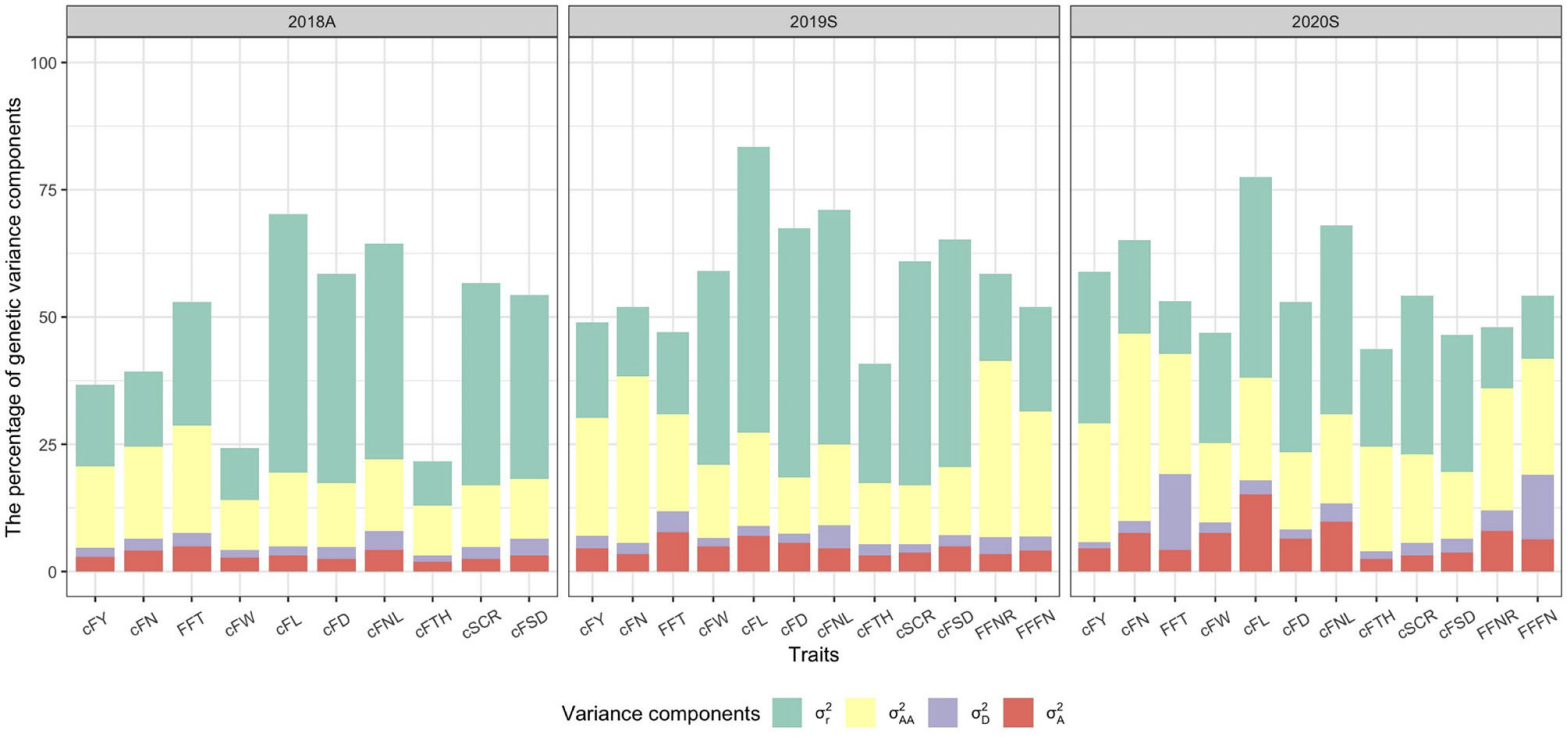
The percentage of genetic variance components in the total variance among traits under the general combining ability (A–D–E) model. $\sigma_{A}^{2}$, $\sigma_{D}^{2}$, $\sigma_{AA}^{2}$, and $\sigma_{r}^{2}$ are additive, dominance, additive-by-additive, and residual genetic variance components, respectively.

Besides, the percentage of genetic variance components in the total variance among traits under the GCA (A–D–E) model was visualized (Figure 4). $\sigma_{A}^{2}$ components accounted for low proportions of the total genetic variance (1.9–15.2%), especially for cFTH in 2018A (1.9%). $\sigma_{D}^{2}$ components also accounted for a lower proportion of the total genetic variance (1.2–14.9%), and only for FFT, FFFN in 2020S had relatively higher dominant variance (12.7–14.9%). $\sigma_{AA}^{2}$ and $\sigma_{r}^{2}$ components accounted for higher proportions of the total genetic variance, respectively (9.7–36.8% for $\sigma_{AA}^{2}$ and 8.6–56.1% for $\sigma_{r}^{2}$).

### The Phenotypic Correlation of DC Population in Three Seasons

The phenotypic correlation of the DC population of each trait in 2018A, 2019S, and 2020S was estimated (Supplementary Figure 3). Most of the traits have a relatively higher correlation in three seasons, especially for cFL traits (*r* = 0.92–0.95). However, the cFTH showed relatively inconsistent correlations among these three seasons, in which the cFTH in 2018A has a weak correlation with the cFTH in 2019S and 2020S (*r* = −0.02–0.21), while cFTH trait in 2019S has a relatively higher correlation with that in 2020S (*r* = 0.63).

### Model Predictive Ability Under Cross-Population Strategy

For the cross-population strategy, there were three validation schemes (Table 3). The NC population in 2020S was the validation group, and the DC population in 2018A, 2019S, and 2020S were the training groups, respectively. As shown in Table 3, when the DC population in 2018A was the training group, the GCA (A) model was enough to obtain the best performance for cFW, cFL, cFNL, cFTH, cSCR, and cFSD, and the GCA (A–D–E) model had the best performance for cFY, cFN, FFT, and cFD. When the DC population in 2019S and 2020S were training groups, the GCA (A–D–E) model performed the best for most of the traits, except for cFL in 2020S and FFNR in both 2019S and 2020S.

**Table 3:** The model predictive ability under the cross-population strategy.

**Validation strategy**	**Trait**	**GCA model**		
		**GCA (A)**	**GCA (A–D)**	**GCA (A–D–E)**
2018A(DC)–>2020S(NC)	cFY	0.621 (0.002) b	0.600 (0.003) c	0.627 (0.002) a
	cFN	0.803 (0.003) c	0.828 (0.003) b	0.850 (0.002) a
	FFT	0.334 (0.003) c	0.388 (0.003) b	0.526 (0.005) a
	cFW	0.749 (0.003) a	0.676 (0.005) c	0.731 (0.003) b
	cFL	0.849 (0.005) a	0.817 (0.005) b	0.835 (0.008) a
	cFD	0.592 (0.005) b	0.593 (0.003) b	0.609 (0.005) a
	cFNL	0.819 (0.003) a	0.781 (0.005) b	0.821 (0.003) a
	cFTH	−0.066 (0.019) a	−0.072 (0.020) a	−0.383 (0.010) b
	cSCR	0.403 (0.022) a	0.379 (0.017) a	0.426 (0.014) a
	cFSD	0.677 (0.007) a	0.602 (0.008) b	0.692 (0.004) a
2019S(DC)–>2020S(NC)	cFY	0.625 (0.001) c	0.637 (0.001) b	0.658 (0.001) a
	cFN	0.813 (0.002) c	0.840 (0.002) b	0.860 (0.002) a
	FFT	0.393 (0.002) c	0.470 (0.001) b	0.602 (0.003) a
	cFW	0.794 (0.005) c	0.807 (0.004) b	0.839 (0.004) a
	cFL	0.831 (0.011) b	0.839 (0.010) ab	0.862 (0.010) a
	cFD	0.602 (0.002) b	0.609 (0.003) b	0.624 (0.004) a
	cFNL	0.830 (0.004) c	0.845 (0.005) b	0.874 (0.005) a
	cFTH	0.386 (0.010) b	0.411 (0.007) b	0.448 (0.013) a
	cSCR	0.452 (0.012) b	0.443 (0.010) b	0.488 (0.014) a
	cFSD	0.668 (0.011) b	0.656 (0.017) b	0.708 (0.008) a
	FFNR	0.811 (0.002) b	0.845 (0.001) a	0.816 (0.004) b
	FFFN	0.746 (0.002) b	0.770 (0.002) a	0.765 (0.007) a
2020S(DC)–>2020S(NC)	cFY	0.623 (0.002) b	0.616 (0.002) c	0.646 (0.002) a
	cFN	0.780 (0.004) c	0.811 (0.004) b	0.839 (0.004) a
	FFT	0.393 (0.001) c	0.485 (0.002) b	0.615 (0.005) a
	cFW	0.817 (0.006) ab	0.803 (0.006) b	0.827 (0.005) a
	cFL	0.821 (0.011) b	0.854 (0.007) a	0.876 (0.004) a
	cFD	0.597 (0.005) b	0.599 (0.007) b	0.635 (0.009) a
	cFNL	0.834 (0.003) b	0.818 (0.005) c	0.868 (0.003) a
	cFTH	0.324 (0.013) b	0.235 (0.022) c	0.378 (0.016) a
	cSCR	0.455 (0.015) b	0.348 (0.033) c	0.537 (0.017) a
	cFSD	0.692 (0.007) b	0.648 (0.014) c	0.727 (0.003) a
	FFNR	0.799 (0.001) c	0.845 (0.001) a	0.831 (0.002) b
	FFFN	0.748 (0.001) c	0.787 (0.001) b	0.825 (0.003) a

*Regarding the GCA model, (A) is an additive model, (A–D) is an additive–dominance model, and (A–D–E) is the additive–dominance–epistasis (additive-by-additive) model. For the cross-population strategy, the NCII population in spring 2020 is the validation group, and the DC population in autumn 2018, spring 2019, and spring 2020 are training groups*.

*The Pearson's correlation coefficient between the observed value and the predicted value is calculated as model predictability. The prediction ability is presented as mean (SD). Different letters indicate a significant difference in the predictive abilities of different models for the same trait. (p < 0.05, Duncan's new multiple range test)*.

*GCA, general combining ability; cFY, commercial fruit yield; cFN, commercial fruit number; FFT, female flowering time; cFW, commercial fruit weight; cFL, commercial fruit length; cFD, commercial fruit diameter; cFNL, commercial fruit neck length; cFTH, commercial fruit flesh thickness; cSCR, commercial seed cavity radius; cFSD, commercial fruit spine density; FFNR, female flower node ratio; FFFN, first female flower node*.

Besides, most of the traits had effective predictive ability under the cross-population strategy, indicating that the GCA models are effective for cross-population prediction, while cSCR and cFTH under the cross-population strategy had relatively worse performance (−0.38 to 0.53).

### The GCA Estimation Result of 81 Inbred Lines Under the Full GCA Model

The estimated GCA information of 81 inbred lines during three seasons is listed in Supplementary Table 4. The result shows that most of the inbred lines in the East Asian and Eurasian groups had higher fruit yield, earlier FFT, and more diverse fruit commercial traits. The GCA effects including additive and additive-by-additive genetic effects are stable genetic effects between generations, and they are essential for evaluating inbred lines. Overall, the estimated GCA information of these 81 inbred lines could provide useful guidance in cucumber breeding practice, which makes the breeding scheme more purposeful.

## Discussion

### The Discussion About GCA Models

The GCA model has been developed by González-Diéguez et al. (2021), which can trace genetic effects of male and female parents (“according to origin”) based on the GCA and SCA theoretical framework. Moreover, it can also estimate the orthogonal variance components. The correlation among the variance components estimated by the GCA (A–D–E) model for cFY (Supplementary Figure 4) shows that the correlation relationship among genetic variance components is weak (*r* ≈ 0), which might be due to its orthogonal prior hypothesis. Orthogonal variance components are essential for the estimation of genetic effects, because the non-orthogonal variance components have overlaps among each other, and genetic effects cannot be estimated accurately. Therefore, the GCA model is a more powerful tool for the estimation of variance components.

Residual genetic effects contain high-order additive effects and interaction (epistatic) effects (*g*_*AAA*_, *g*_*AAAA*_, …), which are important for the genetic structure (González-Diéguez et al., 2021) but are difficult to be estimated separately. In this study, the GCA model without residual genetic effects was also considered. The variance components estimated by the GCA models without residual genetic effects (Supplementary Table 5) showed that the additive and the additive-by-additive effects will have overestimated tendency when residual genetic effects do not exist, though dominance effect and broad-sense heritability may not have obvious changes. The residual genetic effects will be estimated separately when residual genetic effects exist, causing the genetic variance estimation more accurate. However, due to the *a priori* hypothesis that the variance components are orthogonal, the dominant and the residual items are independent and not affected.

Besides, in the GCA model developed by González-Diéguez et al. (2021), the parents come from different populations. In this study, we assumed that both female and male parents are from the same population because cultivated cucumber species have relatively lower genetic diversity and similar genetic structure due to the severe domestication bottleneck events (Qi et al., 2013). Therefore, there were some differences in the details of the GCA models compared to the previous study (González-Diéguez et al., 2021). In addition, this study involved some wild and semi-wild germplasm, which were not suitable for direct breeding of commercial cucumber varieties. However, the significance of including this germplasm for model training was to expand the genetic background of the training population and make the genomic prediction models more robust (Crain et al., 2020).

### Additive and Non-additive Genetic Effects in GCA Models

The additive effect, also called “breeding value,” is the accumulation of genotype values of minor genes (Hayes et al., 2009). An additive effect is essential for a breeding program because it is stable and accumulable between generations. Non-additive effects, like dominance and additive-by-additive effects, are also important to capture more genetic variance (Varona et al., 2018). Although non-additive models are transient and highly dependent on the heterozygosity of the population, F_1_ hybrids are final cultivars in a single-cross breeding system, thus non-additive effects can provide more accurate information for the prediction of F_1_ hybrid performance, and the performance of three GCA models under the five-fold cross-validation strategy verified this conclusion (Table 1). Some previous studies have reported that models containing non-additive effects perform better than pure additive models in some cases (Munoz et al., 2014; Dias et al., 2018; Wu et al., 2019), especially for the traits with lower heritability (Liu et al., 2018).

The proportion of each genetic variance to the total genetic variance reflects the degree of control on each genetic effect of traits. In this study, the SCA effect ($\sigma_{D}^{2}$) accounts for only a small part (1.2–14.9%) of the total genetic variance for most of the traits (Figure 4) compared with the GCA effect (20.4–81.4%). This estimation result indicates that the GCA effect plays a leading role in explaining phenotypic variance in cucumber. Therefore, breeding excellent inbred lines is very important for selecting superior cucumber hybrid varieties, and Supplementary Table 4 shows the valuable GCA information for 81 cucumber inbred lines.

Although year/season effects have important impacts on breeding work, we did not handle these effects in the model, as we preferred to analyze and compare the changes of genetic effects in different seasons/years. But the phenotypic correlation of the DC population among three seasons was calculated to check whether there were any obvious year/seasonal effects. It was stated as G × E effects (where G signifies genotypes and E signifies environments). Most of the traits have a relatively higher correlation in three seasons indicating that there were no obvious G × E for these traits.

### The Comparison of Two Model Validation Strategies

In this study, two model validation strategies were used, which were five-fold cross-validation and cross-population strategies. In the previous studies, *k*-fold cross-validation and leave-one-out cross-validation are the two main methods for model predictive ability estimation (Wu et al., 2019; Cui et al., 2020), which are similar to the five-fold cross-validation strategy in this study. The cross-validation strategy provides an effective tool for checking overfitting issues among three GCA models. For instance, the cFL in 2018A, the GCA (A) model is enough to obtain the best performance, since dominance and additive-by-additive genetic components occupy a relatively small proportion of the total genetic variance (23.1%), thus more complex GCA models are unnecessary.

For the five-fold cross-validation strategy, the model predictive ability represents the reliability estimation of prediction results. In general, the predictive ability has a positive correlation with trait heritability (Combs and Bernardo, 2013), indicating that the higher predictive ability means higher trait heritability. Furthermore, the higher trait heritability means the higher proportion of genetic variance explained by the genomic prediction model to the total variance, resulting in more reliable estimates for untested inbreds and combinations. For example, the predictive ability of cFL is 0.90–0.95 under the GCA (A–D–E) model, and the corresponding heritability is 0.70–0.83. In fact, cFL is more stable under different environments compared with yield-related traits. Therefore, traits with high predictive ability will provide more precise references to practical breedings.

The cross-population strategy is cross-population validation in the same or different seasons. When the training group and the validation group are in the same season, the environmental factor is similar, and the model performance mainly depends on the genetic similarity between populations (Sverrisdottir et al., 2018; Edwards et al., 2019). More complex models can capture a higher genetic variance proportion of the training group for explaining the variance in the validation group. Thus, more complex models have better performance (Table 3, 2020S(DC)–>2020S(NC) validation strategy).

When the training group and the validation groups are in a different year, especially in different seasons, not only the genetic similarity between populations but also environmental factors may affect the model performance. Although GCA (A–D) and GCA (A–D–E) models can catch non-additive genetic effects, like dominance and additive-by-additive variance components, these non-additive effects are transient and highly dependent on the population structure and specific environment. The additive effect is relatively stable among populations but the additive effect is reduced in the non-additive models, especially in the GCA (A–D–E) model (Supplementary Table 3). Therefore, the GCA (A) model is enough for cFW, cFL, cFNL, cFTH, cSCR, and cFSD under the “2018A(DC)–>2020S(NC)” validation strategy (Table 3).

### The Comparison Between GCA Models and Traditional Combining Ability Estimation Methods

Combining ability estimation has always been an important content in plant breeding, which provides a valuable reference for the breeding of inbreds and hybrids (Lv et al., 2012). There are some studies on combining ability estimation in cucumber breeding (López-Sesé and Staub, 2002; Golabadi et al., 2015; Ene et al., 2019), where rigorous field experimental design and phenotypic variance decomposition were used to estimate GCA and SCA. In these studies, SCA has a substantial contribution to some traits. For example, FL and FD have high proportions of variance of SCA in the study by Golabadi et al. (2015), but the variance of SCA for these traits may be overestimated.

In this study, the genetic relationship matrices based on high-density markers and the phenotypes of the training population were used for model training, and genetic effects were estimated accurately based on the assumption of orthogonal genetic variance. The results show that for all investigated traits, the SCA (dominance effect) only accounts for a small part of the total variance (1.2–14.9%), and the GCA accounts for the main part of the total variance (20.4–81.4%). Besides, in actual breeding practice, the heterosis of cucumber is not obvious in most cases (Weng, 2021), indicating that the proportion of non-additive effects (SCA) is low. Therefore, the GCA models in this study may be more accurate in combining ability estimation compared with traditional methods.

In addition, the GCA model can predict the GCA of inbred lines, which have genotypes but not involved in field trials. Moreover, the GCA models do not have strict requirements for the design of the training population, though more representative training groups will have more reliable results. These advantages can make combining ability estimation more efficient and then saving the costs in breeding.

### Opportunities and Challenges of Genomic Prediction Applied to Cucumber Breeding

The traditional hybrid breeding scheme limits the efficiency of cucumber breeding, as potential hybrids that need to be tested will increase exponentially when parents increase linearly. The genomic prediction could perform large-scale accurate prediction with a smaller training group, which improves the selection efficiency significantly and meet the increasing demand for breeding tasks. Besides, the cucumber genome is relatively small (~400 Mbp) (Weng, 2021), and fewer molecular markers can achieve high marker density, which is an advantage of low cost for genomic prediction applied to cucumber breeding.

Although genomic prediction has broad application prospects in cucumber breeding, there are some challenges that need to be considered. First, some fresh market cucumber species should be planted in a greenhouse, but the greenhouse area may limit the training population size. Second, some cucumber agronomic traits are important but difficult to quantify, such as fruit crispness, fruit aroma, fruit skin color consistency, etc. Therefore, more in-depth studies are needed to quantify these traits to support the application of genomic prediction. Regarding further studies, stress resistance and fruit nutritional quality traits of cucumber will be considered in genomic prediction to make the breeding scheme more practicable.

## Data Availability Statement

The datasets presented in this study can be found in online repositories. The names of the repository/repositories and accession number(s) can be found at: NCBI BioProject accession: PRJNA741624.

## Author Contributions

CL: data curation, investigation, methodology, software, writing—original draft, writing—review, and editing. XL: data curation, investigation, methodology, writing—review, and editing. YH: funding acquisition, project administration, and supervision. XW and YD: methodology, writing—review, and editing. HM: funding acquisition, project administration, supervision, writing—review, and editing. ZC: conceptualization, project administration, supervision, writing—original draft, writing—review, and editing. All authors contributed to the article and approved the submitted version.

## Conflict of Interest

The authors declare that the research was conducted in the absence of any commercial or financial relationships that could be construed as a potential conflict of interest.

## Publisher's Note

All claims expressed in this article are solely those of the authors and do not necessarily represent those of their affiliated organizations, or those of the publisher, the editors and the reviewers. Any product that may be evaluated in this article, or claim that may be made by its manufacturer, is not guaranteed or endorsed by the publisher.
