# Supplementary material for: Genomic Prediction and the Practical Breeding of 12 Quantitative-Inherited Traits in Cucumber (Cucumis sativus L.)
Source: Front Plant Sci. 2021 Aug 24;12:729328. doi: 10.3389/fpls.2021.729328 (PMC8421847; doi:10.3389/fpls.2021.729328)
Supplement: Supplementary file 1 [file Data_Sheet_1.zip › Supplementary Figure 3.PDF]

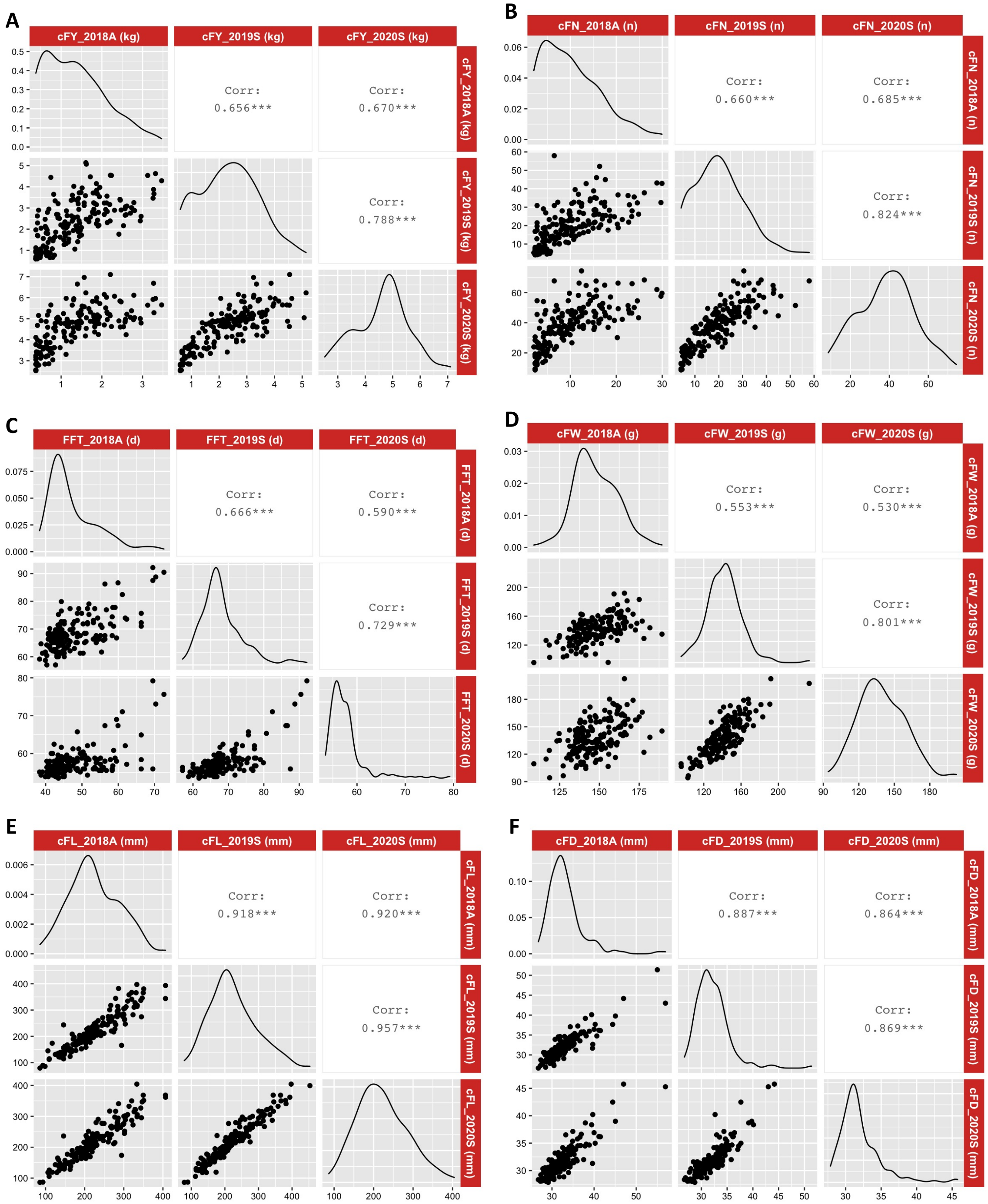
A
cFY_2018A (kg)
cFY_2019S (kg)
cFY_2020S (kg)
Corr: 0.656***
Corr: 0.670***
Corr: 0.788***
B
cFN_2018A (n)
cFN_2019S (n)
cFN_2020S (n)
Corr: 0.660***
Corr: 0.685***
Corr: 0.824***
C
FFT_2018A (d)
FFT_2019S (d)
FFT_2020S (d)
Corr: 0.666***
Corr: 0.590***
Corr: 0.729***
D
cFW_2018A (g)
cFW_2019S (g)
cFW_2020S (g)
Corr: 0.553***
Corr: 0.530***
Corr: 0.801***
E
cFL_2018A (mm)
cFL_2019S (mm)
cFL_2020S (mm)
Corr: 0.918***
Corr: 0.920***
Corr: 0.957***
F
cFD_2018A (mm)
cFD_2019S (mm)
cFD_2020S (mm)
Corr: 0.887***
Corr: 0.864***
Corr: 0.869***

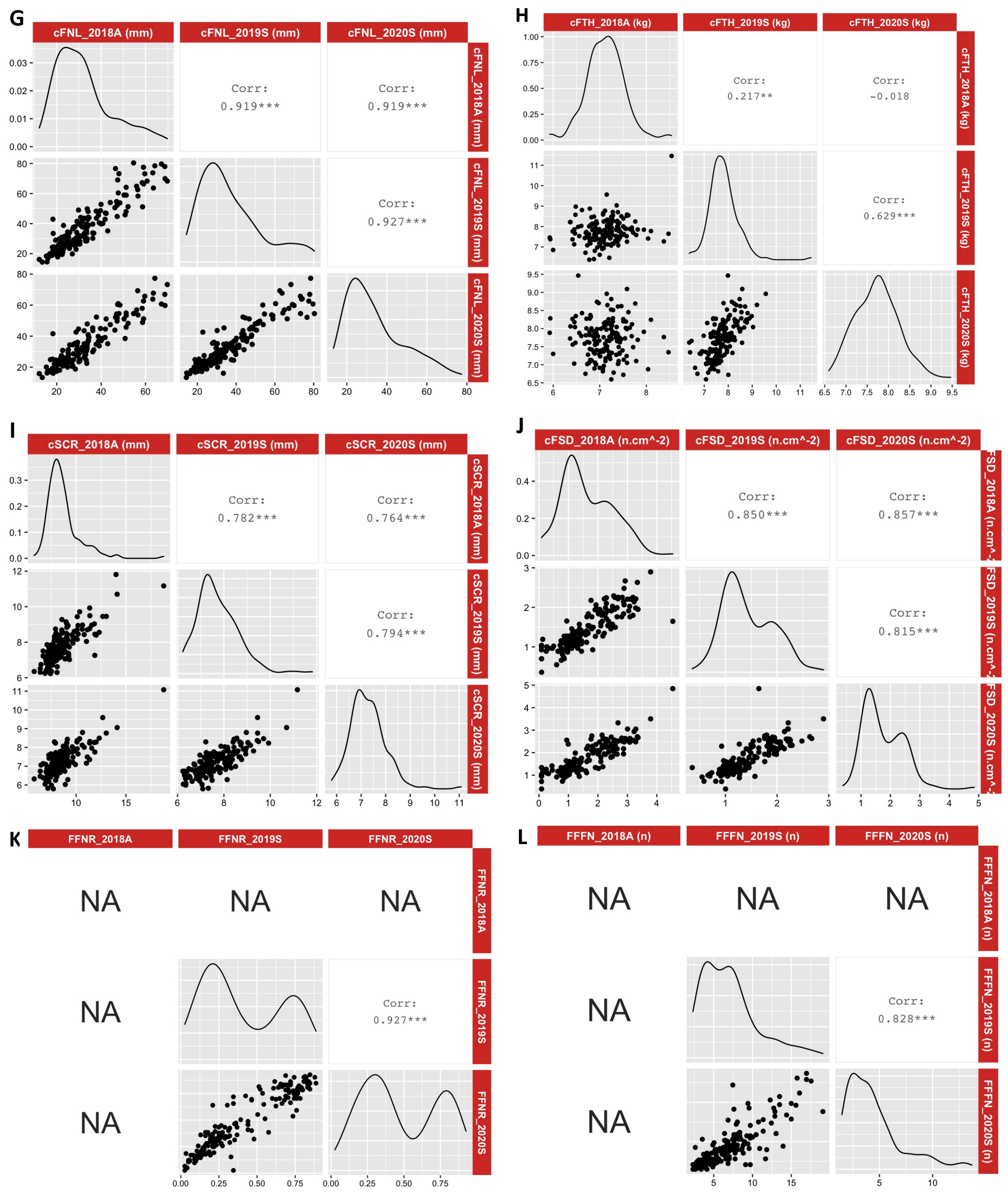

Supplementary Figure 3. The correlation relationship of phenotypic data of DC population in three seasons. 2018A: autumn 2018; 2019S: spring 2019; 2020S: spring 2020. A-L are cFY, cFN, FFT, cFW, cFL, cFD, cFNL, cFFT, cSCR, cFSD, FFNR, and FFFN traits respectively. The Pearson’s correlation coefficient is used to show the correlation relationship of traits among seasons (***: $p < 0.001$; **: $p < 0.01$; *: $p < 0.05$).
