# Supplementary material for: Genomic Prediction and the Practical Breeding of 12 Quantitative-Inherited Traits in Cucumber (Cucumis sativus L.)
Source: Front Plant Sci. 2021 Aug 24;12:729328. doi: 10.3389/fpls.2021.729328 (PMC8421847; doi:10.3389/fpls.2021.729328)
Supplement: Supplementary file 1 [file Data_Sheet_1.zip › Supplementary Figure 4.PDF]

A

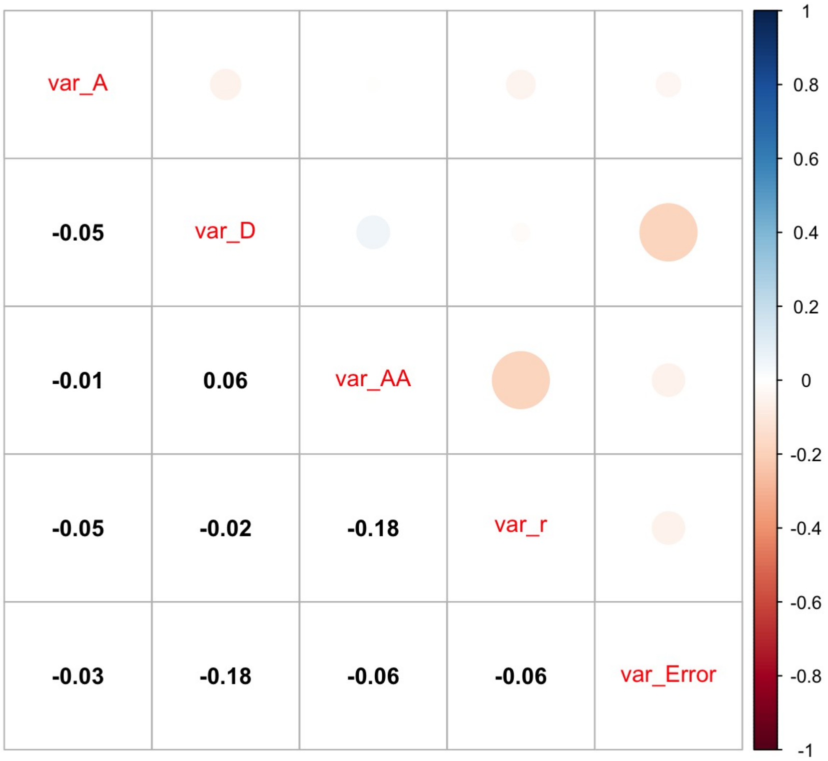

var_A
-0.05
var_D
-0.01
0.06
var_AA
-0.05
-0.02
-0.18
var_r
-0.03
-0.18
-0.06
-0.06
var_Error
1
0.8
0.6
0.4
0.2
0
-0.2
-0.4
-0.6
-0.8
-1

B

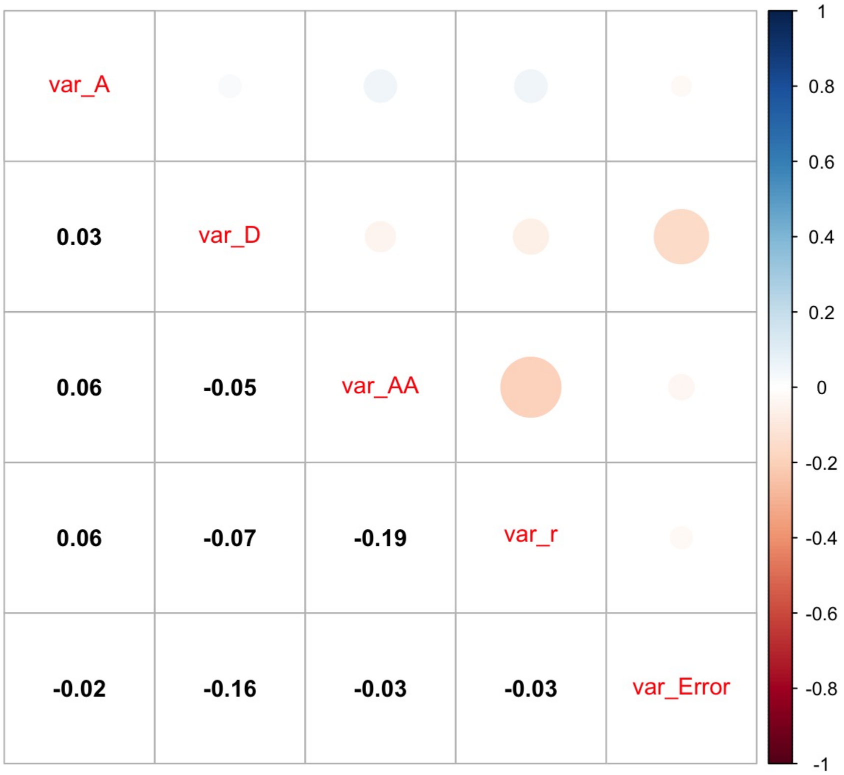

var_A
0.03
var_D
0.06
-0.05
var_AA
0.06
-0.07
-0.19
var_r
-0.02
-0.16
-0.03
-0.03
var_Error
1
0.8
0.6
0.4
0.2
0
-0.2
-0.4
-0.6
-0.8
-1

C

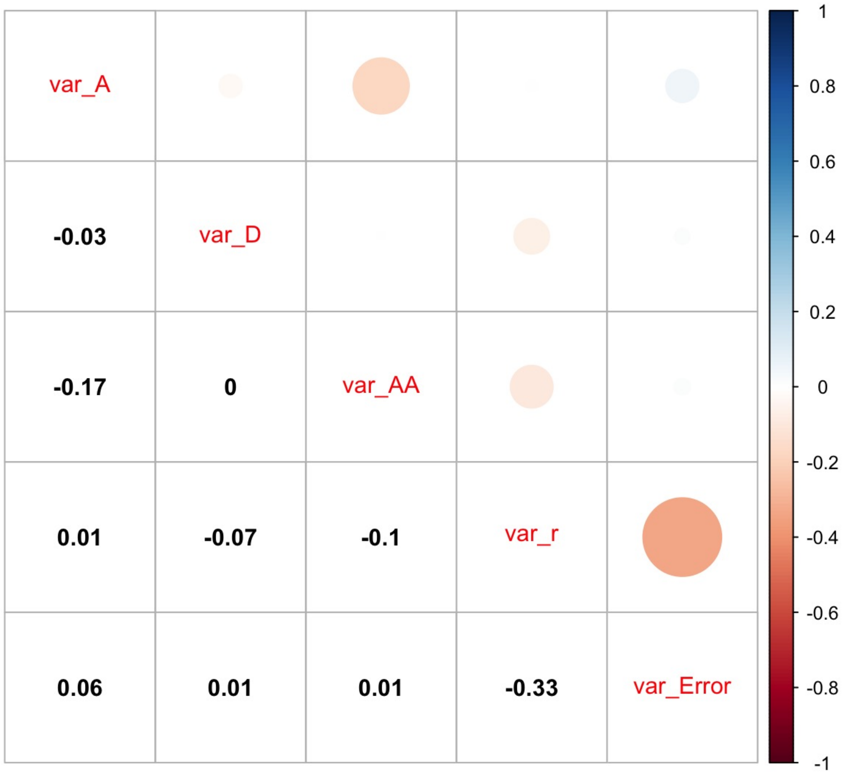

Supplementary Figure 4. The-posteriori correlations among estimated variance components. A-C are the estimated variance components of cFY traits in spring 2018, spring 2019, and spring 2020 respectively using full GCA -model. var_r is the variance component of the "residual genetic" r, var_Error is the variance component of the residual item.
