# Supplementary material for: Genomic Prediction and the Practical Breeding of 12 Quantitative-Inherited Traits in Cucumber (Cucumis sativus L.)
Source: Front Plant Sci. 2021 Aug 24;12:729328. doi: 10.3389/fpls.2021.729328 (PMC8421847; doi:10.3389/fpls.2021.729328)
Supplement: Supplementary file 1 [file Data_Sheet_1.zip › Supplementary File 3.PDF]

R functions for model training and validation

- Functions for model training and validation

Functions for model training and validation

```
library(stringr)
library(BGLR)
library(ggplot2)

# GCA model (contrain residual genetic r effects)
GCA_models.r <- function(y,hyb,X,all_hyb,model="BRR",model_type){
  #the full model
  #y = u+T1*g(A)+T2*g(A)+g(D)+T1g(AA)+T2g(AA)+g(A1A2)+T1r+T2r+e
  #y: phenotypic data
  #hyb: hybrids name
  #X: inbred line genotype matrix
  #all_hyb: inbred lines name
  #model: bayesian model type (bayesian ridge regression model is default)
  #model_type: "A": GCA(A) model; "A-D": GCA(A-D) model; "A-D-E": GCA (A-D-E) mode
l.

  ##GCA relationship matrix construction
  n0 <- nrow(X)     #number of inbred lines
  nc <- length(y) ##number of tested hybrids
  nsnp <- ncol(X)   #number of SNPs

  p1=rep(NA,nsnp)
  for (i in 1:nsnp){
    p1[i] <- mean(X[,i])
  }
  q1=1-p1

  #create incidence matrices Z1
  Z1 <- X
  for (i in 1:nsnp){
    Z1[,i]=X[,i]-p1[i]
  }

  G_GCA <- (Z1%*%t(Z1))/sum(p1*q1)### G_A for inbred lines

  #DOMINANCE GENOMIC RELATIONSHIP
  Parent_female <- rep(NA,length(y))
  Parent_male <- rep(NA,length(y))
  for (i in 1:length(y)) {
    Parent_female[i] <- str_split(hyb[i],"×")[[1]][1]
```

```
  Parent_male[i] <- str_split(hyb[i],"×")[[1]][2]
}

# incidence matrix of dominant effects W
W=matrix(NA,ncol=nsnp,nrow=nc)
for (i in 1:nc){
  w1.i <- which(all_hyb==Parent_female[i])
  w2.i <- which(all_hyb==Parent_male[i])
  m1 <- X[w1.i,]
  m2 <- X[w2.i,]
  val=-2*((-1)**(m1+m2))*(q1*m1+p1*(1-m1))*(q1*m2+p1*(1-m2))
  W[i,]=val
}
rownames(W)= hyb
# Dominance Relationship D
D <- W%**%t(W)/mean(diag(W%**%t(W))) #scaled by trace of matrix

#AxA Epistasis within-line -  G_AA(1,1) for female and male parents
EAA <- G_GCA * G_GCA
EAA <- EAA/mean(diag(EAA)) #scaled by trace of matrix

#incidence matrices construction (T1, T2)
T1 <- matrix(rep(0,length(y)*nrow(X)),nrow = length(y))
colnames(T1) <- rownames(X)
rownames(T1) <- Parent_female
for (i in 1:nrow(T1)) {
  hyb.f <- Parent_female[i]
  w.i <- which(rownames(X)==hyb.f)
  T1[i,w.i] <- 1
}

T2 <- matrix(rep(0,length(y)*nrow(X)),nrow = length(y))
colnames(T2) <- rownames(X)
rownames(T2) <- Parent_male
for (i in 1:nrow(T2)) {
  hyb.m <- Parent_male[i]
  w.i <- which(rownames(X)==hyb.m)
  T2[i,w.i] <- 1
}

## model training
G_A1 <- T1 %*% G_GCA
G_A2 <- T2 %*% G_GCA
G_AM <- T1 %*% G_GCA + T2 %*% G_GCA

EAAs1 <- T1 %*% EAA
EAAs2 <- T2 %*% EAA
EAAM <- T1 %*% EAA + T2 %*% EAA

rM <- T1 %*% diag(n0) + T2 %*% diag(n0)

if(model_type=="A"){
  ETAcomp <- list(G_AM2=list(X=G_AM,model=model,saveEffects=T),
                  G_r=list(X=rM,model=model,saveEffects=T))
```

```
}else if(model_type=="A-D"){
  ETAcomp <- list(G_AM2=list(X=G_AM,model=model,saveEffects=T),
                  G_D=list(X=D,model=model,saveEffects=T),
                  G_r=list(X=rM,model=model,saveEffects=T))
}else if(model_type=="A-D-E"){
  ETAcomp <- list(G_AM2=list(X=G_AM,model=model,saveEffects=T),
                  G_D=list(X=D,model=model,saveEffects=T),
                  G_AAM=list(X=EAAM,model=model,saveEffects=T),
                  G_r=list(X=rM,model=model,saveEffects=T))
}

#k-folds cross-validation
nrep <- 20;folds <- 5
res_CV <- matrix(rep(NA,nrep*folds),ncol = 5)
colnames(res_CV) <- paste(rep("k"),1:folds,sep = "_")
rownames(res_CV) <- paste(rep("rep"),1:nrep,sep = "_")

y2 <- y
val <- sample(1:folds,length(y),replace = T)
for (n in 1:nrep) {
  cat("The",n,"th cycles:\n")
  for (m in 1:folds) {
    k <- which(val==m)
    #cat("when k =",m,":\n")
    y[k] <- NA
    fm.m <-BGLR(y=y,ETA=ETAcomp, nIter=30000, burnIn=10000,verbose = FALSE)
    pred.m <- fm.m$yHat[k]
    obs.m <- y2[k]
    res_CV[n,m] <- cor(pred.m,obs.m)
    y <- y2
  }
}
res_CV2 <- apply(res_CV,1,mean)

#variance component
setwd("~/GBLUP")
fm <- BGLR(y=y,ETA=ETAcomp,nIter=30000,burnIn=10000,verbose=F)
res_Varcomp <- matrix(rep(NA,5*2),ncol = 2)
colnames(res_Varcomp) <- c("varB","SD.varB")
rownames(res_Varcomp) <- c("G_AM","G_D","G_EAAM","G_r","Error")
if(model_type=="A"){
  res_Varcomp[1,1] <- fm$ETA$G_AM2$varB;res_Varcomp[1,2] <- fm$ETA$G_AM2$SD.varB
  res_Varcomp[4,1] <- fm$ETA$G_r$varB;res_Varcomp[4,2] <- fm$ETA$G_r$SD.varB
  res_Varcomp[5,1] <- fm$varE;res_Varcomp[5,2] <- fm$SD.varE
}else if(model_type=="A-D"){
  res_Varcomp[1,1] <- fm$ETA$G_AM2$varB;res_Varcomp[1,2] <- fm$ETA$G_AM2$SD.varB
  res_Varcomp[2,1] <- fm$ETA$G_D$varB;res_Varcomp[2,2] <- fm$ETA$G_D$SD.varB
  res_Varcomp[4,1] <- fm$ETA$G_r$varB;res_Varcomp[4,2] <- fm$ETA$G_r$SD.varB
  res_Varcomp[5,1] <- fm$varE;res_Varcomp[5,2] <- fm$SD.varE
}else if(model_type=="A-D-E"){
  res_Varcomp[1,1] <- fm$ETA$G_AM2$varB;res_Varcomp[1,2] <- fm$ETA$G_AM2$SD.varB
  res_Varcomp[2,1] <- fm$ETA$G_D$varB;res_Varcomp[2,2] <- fm$ETA$G_D$SD.varB
  res_Varcomp[3,1] <- fm$ETA$G_AAM$varB;res_Varcomp[3,2] <- fm$ETA$G_AAM$SD.varB
  res_Varcomp[4,1] <- fm$ETA$G_r$varB;res_Varcomp[4,2] <- fm$ETA$G_r$SD.varB
```

```
    res_Varcomp[5,1] <- fm$varE;res_Varcomp[5,2] <- fm$SD.varE
  }
  #model fitness DIC
  res_DIC <- fm$fit$DIC
  ress <- list(res_DIC,res_CV2,res_Varcomp)
  names(ress) <- c("model fitness (DIC)","5-folds cross validation","Variance comp
onents")
  return(ress)
}
```
