# Supplementary material for: Genomic Prediction and the Practical Breeding of 12 Quantitative-Inherited Traits in Cucumber (Cucumis sativus L.)
Source: Front Plant Sci. 2021 Aug 24;12:729328. doi: 10.3389/fpls.2021.729328 (PMC8421847; doi:10.3389/fpls.2021.729328)
Supplement: Supplementary file 1 [file Data_Sheet_1.zip › Supplementary Table 3.DOCX]

Supplementary Table 3. The estimated posterior of genetic variance components, broad-sense heritability (*H^2^*) under three GCA models for 11 traits (except for cFY traits) in three seasons

| Trait | Season | Model | Variance components | | | | | *H^2^* |
| --- | --- | --- | --- | --- | --- | --- | --- | --- |
|  |  |  | $\sigma_{A}^{2}$ | $\sigma_{D}^{2}$ | $\sigma_{AA}^{2}$ | $\sigma_{r}^{2}$ | $\sigma_{\varepsilon}^{2}$ |  |
| cFN | 2018A | GCA (A) | 1.899(1.467) |  |  | 8.360(3.737) | 20.086(2.263) | 0.338 |
|  | 2018A | GCA (A-D) | 2.253(1.935) | 0.838(0.650) |  | 6.821(3.413) | 18.749(2.289) | 0.346 |
|  | 2018A | GCA (A-D-E) | 1.274(1.751) | 0.699(0.587) | 5.629(4.298) | 4.554(2.424) | 18.769(2.284) | 0.393 |
|  | 2019S | GCA (A) | 9.302(6.445) |  |  | 21.003(10.937) | 34.142(3.876) | 0.470 |
|  | 2019S | GCA (A-D) | 8.194(5.178) | 2.094(1.116) |  | 17.379(10.197) | 31.087(3.898) | 0.471 |
|  | 2019S | GCA (A-D-E) | 2.253(2.541) | 1.453(0.890) | 21.131(10.786) | 8.866(4.681) | 31.032(3.768) | 0.521 |
|  | 2020S | GCA (A) | 25.627(12.396) |  |  | 26.256(8.445) | 47.589(4.707) | 0.522 |
|  | 2020S | GCA (A-D) | 42.557(16.972) | 2.480(1.111) |  | 20.296(6.326) | 32.781(3.562) | 0.666 |
|  | 2020S | GCA (A-D-E) | 7.198(5.467) | 2.174(0.954) | 34.698(17.562) | 17.363(5.839) | 32.860(3.552) | 0.652 |
| FFT | 2018A | GCA (A) | 4.149(2.239) |  |  | 9.145(4.236) | 15.628(1.753) | 0.460 |
|  | 2018A | GCA (A-D) | 3.576(1.979) | 0.885(0.497) |  | 8.370(3.967) | 14.133(1.726) | 0.476 |
|  | 2018A | GCA (A-D-E) | 1.506(1.123) | 0.780(0.482) | 6.336(4.156) | 7.279(3.586) | 14.198(1.753) | 0.528 |
|  | 2019S | GCA (A) | 2.748(1.395) |  |  | 5.381(2.476) | 16.026(1.827) | 0.337 |
|  | 2019S | GCA (A-D) | 3.875(2.164) | 1.047(0.636) |  | 4.385(1.915) | 12.215(1.527) | 0.432 |
|  | 2019S | GCA (A-D-E) | 1.778(1.632) | 0.949(0.622) | 4.416(2.922) | 3.738(1.752) | 12.299(1.534) | 0.469 |
|  | 2020S | GCA (A) | 1.219(0.706) |  |  | 1.110(0.360) | 6.164(0.579) | 0.274 |
|  | 2020S | GCA (A-D) | 1.768(0.833) | 1.173(0.489) |  | 0.860(0.290) | 3.422(0.424) | 0.526 |
|  | 2020S | GCA (A-D-E) | 0.305(0.267) | 1.049(0.399) | 1.647(0.719) | 0.729(0.251) | 3.291(0.414) | 0.531 |
| cFW | 2018A | GCA (A) | 5.929(4.008) |  |  | 25.221(11.192) | 119.397(13.471) | 0.207 |
|  | 2018A | GCA (A-D) | 4.040(2.904) | 2.667(1.395) |  | 21.394(9.046) | 114.002(13.219) | 0.198 |
|  | 2018A | GCA (A-D-E) | 4.162(4.716) | 2.495(1.574) | 14.745(8.940) | 15.724(7.751) | 115.325(13.851) | 0.244 |
|  | 2019S | GCA (A) | 14.880(12.126) |  |  | 89.829(34.595) | 84.630(9.755) | 0.553 |
|  | 2019S | GCA (A-D) | 7.629(4.774) | 4.812(2.994) |  | 86.351(30.817) | 82.342(9.865) | 0.545 |
|  | 2019S | GCA (A-D-E) | 9.829(8.510) | 3.350(1.616) | 28.677(16.795) | 76.490(35.120) | 82.349(9.759) | 0.590 |
|  | 2020S | GCA (A) | 34.675(20.114) |  |  | 42.199(13.052) | 93.903(9.145) | 0.450 |
|  | 2020S | GCA (A-D) | 33.188(31.573) | 4.169(1.741) |  | 41.330(14.730) | 87.755(8.428) | 0.473 |
|  | 2020S | GCA (A-D-E) | 12.757(14.282) | 3.537(1.711) | 25.854(11.448) | 36.224(11.068) | 88.624(8.770) | 0.469 |
| cFL | 2018A | GCA (A) | 116.341(88.890) |  |  | 1317.062(502.030) | 714.179(81.286) | 0.667 |
|  | 2018A | GCA (A-D) | 89.845(51.657) | 53.392(24.964) |  | 1239.323(415.776) | 662.973(80.390) | 0.676 |
|  | 2018A | GCA (A-D-E) | 71.491(39.232) | 39.176(17.785) | 323.908(217.928) | 1137.771(441.127) | 666.944(78.250) | 0.702 |
|  | 2019S | GCA (A) | 157.179(99.665) |  |  | 1533.455(518.725) | 466.972(52.587) | 0.784 |
|  | 2019S | GCA (A-D) | 97.912(53.647) | 63.471(29.168) |  | 1604.889(574.031) | 413.966(52.677) | 0.810 |
|  | 2019S | GCA (A-D-E) | 175.493(149.928) | 50.468(23.593) | 461.408(341.775) | 1413.979(571.856) | 417.876(52.015) | 0.834 |
|  | 2020S | GCA (A) | 634.717(273.590) |  |  | 454.310(138.545) | 335.845(32.892) | 0.764 |
|  | 2020S | GCA (A-D) | 265.957(175.207) | 33.875(11.506) |  | 576.137(177.894) | 297.143(32.288) | 0.747 |
|  | 2020S | GCA (A-D-E) | 203.046(157.841) | 35.867(19.565) | 269.667(151.293) | 524.470(149.805) | 299.016(32.339) | 0.776 |
| cFD | 2018A | GCA (A) | 0.587(0.400) |  |  | 6.088(2.210) | 5.737(0.651) | 0.538 |
|  | 2018A | GCA (A-D) | 0.399(0.227) | 0.324(0.162) |  | 5.710(1.983) | 5.055(0.600) | 0.560 |
|  | 2018A | GCA (A-D-E) | 0.306(0.213) | 0.281(0.163) | 1.549(1.055) | 5.052(2.060) | 5.097(0.608) | 0.585 |
|  | 2019S | GCA (A) | 0.339(0.232) |  |  | 3.502(1.197) | 2.332(0.264) | 0.622 |
|  | 2019S | GCA (A-D) | 0.200(0.111) | 0.163(0.098) |  | 3.486(1.201) | 2.130(0.258) | 0.644 |
|  | 2019S | GCA (A-D-E) | 0.372(0.482) | 0.127(0.062) | 0.726(0.388) | 3.224(1.344) | 2.143(0.260) | 0.675 |
|  | 2020S | GCA (A) | 0.441(0.347) |  |  | 1.117(0.309) | 1.703(0.166) | 0.478 |
|  | 2020S | GCA (A-D) | 0.426(0.263) | 0.073(0.036) |  | 1.052(0.300) | 1.537(0.157) | 0.502 |
|  | 2020S | GCA (A-D-E) | 0.212(0.186) | 0.055(0.024) | 0.497(0.254) | 0.963(0.262) | 1.539(0.153) | 0.529 |
| cFNL | 2018A | GCA (A) | 5.193(2.890) |  |  | 46.391(16.145) | 35.983(4.340) | 0.589 |
|  | 2018A | GCA (A-D) | 7.218(7.431) | 3.694(2.331) |  | 41.377(15.194) | 31.356(4.174) | 0.625 |
|  | 2018A | GCA (A-D-E) | 3.721(2.478) | 3.333(2.137) | 12.427(6.641) | 37.362(15.423) | 31.516(4.158) | 0.643 |
|  | 2019S | GCA (A) | 9.278(5.418) |  |  | 65.756(22.692) | 45.853(5.282) | 0.621 |
|  | 2019S | GCA (A-D) | 7.793(5.370) | 6.829(3.481) |  | 60.758(20.724) | 33.939(4.477) | 0.690 |
|  | 2019S | GCA (A-D-E) | 5.398(4.017) | 5.468(2.902) | 18.836(10.718) | 54.970(20.010) | 34.697(4.502) | 0.709 |
|  | 2020S | GCA (A) | 29.176(16.416) |  |  | 42.882(12.829) | 44.598(4.498) | 0.618 |
|  | 2020S | GCA (A-D) | 35.596(26.472) | 5.064(2.670) |  | 39.319(14.637) | 35.181(4.022) | 0.695 |
|  | 2020S | GCA (A-D-E) | 11.000(7.064) | 3.986(2.283) | 19.557(9.408) | 41.553(11.734) | 35.922(4.134) | 0.679 |
| cFTH | 2018A | GCA (A) | 0.008(0.009) |  |  | 0.025(0.012) | 0.131(0.015) | 0.206 |
|  | 2018A | GCA (A-D) | 0.005(0.004) | 0.003(0.002) |  | 0.021(0.010) | 0.127(0.016) | 0.189 |
|  | 2018A | GCA (A-D-E) | 0.003(0.003) | 0.002(0.002) | 0.016(0.010) | 0.014(0.007) | 0.127(0.015) | 0.220 |
|  | 2019S | GCA (A) | 0.012(0.008) |  |  | 0.079(0.030) | 0.179(0.020) | 0.338 |
|  | 2019S | GCA (A-D) | 0.014(0.013) | 0.008(0.005) |  | 0.075(0.029) | 0.165(0.020) | 0.371 |
|  | 2019S | GCA (A-D-E) | 0.009(0.008) | 0.006(0.004) | 0.034(0.023) | 0.066(0.028) | 0.167(0.020) | 0.407 |
|  | 2020S | GCA (A) | 0.011(0.009) |  |  | 0.062(0.020) | 0.121(0.012) | 0.376 |
|  | 2020S | GCA (A-D) | 0.014(0.013) | 0.004(0.003) |  | 0.060(0.020) | 0.113(0.011) | 0.409 |
|  | 2020S | GCA (A-D-E) | 0.005(0.005) | 0.003(0.003) | 0.041(0.023) | 0.038(0.014) | 0.112(0.012) | 0.437 |
| cSCR | 2018A | GCA (A) | 0.090(0.058) |  |  | 0.868(0.304) | 0.843(0.098) | 0.532 |
|  | 2018A | GCA (A-D) | 0.053(0.037) | 0.050(0.024) |  | 0.795(0.279) | 0.749(0.087) | 0.545 |
|  | 2018A | GCA (A-D-E) | 0.042(0.027) | 0.042(0.024) | 0.212(0.142) | 0.690(0.260) | 0.753(0.089) | 0.567 |
|  | 2019S | GCA (A) | 0.034(0.026) |  |  | 0.241(0.090) | 0.210(0.023) | 0.568 |
|  | 2019S | GCA (A-D) | 0.016(0.010) | 0.011(0.005) |  | 0.233(0.082) | 0.200(0.023) | 0.564 |
|  | 2019S | GCA (A-D-E) | 0.019(0.018) | 0.008(0.004) | 0.060(0.031) | 0.226(0.088) | 0.202(0.024) | 0.608 |
|  | 2020S | GCA (A) | 0.020(0.017) |  |  | 0.094(0.024) | 0.140(0.014) | 0.449 |
|  | 2020S | GCA (A-D) | 0.011(0.008) | 0.005(0.002) |  | 0.099(0.024) | 0.114(0.012) | 0.504 |
|  | 2020S | GCA (A-D-E) | 0.008(0.005) | 0.006(0.003) | 0.043(0.028) | 0.077(0.022) | 0.113(0.012) | 0.542 |
| cFSD | 2018A | GCA (A) | 0.031(0.029) |  |  | 0.176(0.061) | 0.229(0.027) | 0.474 |
|  | 2018A | GCA (A-D) | 0.017(0.011) | 0.017(0.010) |  | 0.174(0.063) | 0.202(0.025) | 0.508 |
|  | 2018A | GCA (A-D-E) | 0.014(0.011) | 0.015(0.010) | 0.052(0.030) | 0.161(0.065) | 0.203(0.026) | 0.543 |
|  | 2019S | GCA (A) | 0.008(0.005) |  |  | 0.071(0.025) | 0.055(0.006) | 0.589 |
|  | 2019S | GCA (A-D) | 0.004(0.003) | 0.004(0.002) |  | 0.069(0.023) | 0.049(0.006) | 0.613 |
|  | 2019S | GCA (A-D-E) | 0.007(0.008) | 0.003(0.002) | 0.019(0.013) | 0.063(0.024) | 0.049(0.006) | 0.655 |
|  | 2020S | GCA (A) | 0.015(0.010) |  |  | 0.067(0.018) | 0.127(0.012) | 0.393 |
|  | 2020S | GCA (A-D) | 0.016(0.012) | 0.007(0.004) |  | 0.064(0.017) | 0.115(0.012) | 0.432 |
|  | 2020S | GCA (A-D-E) | 0.008(0.005) | 0.006(0.004) | 0.029(0.016) | 0.059(0.017) | 0.117(0.013) | 0.464 |
| FFNR | 2019S | GCA (A) | 0.005(0.003) |  |  | 0.009(0.004) | 0.015(0.002) | 0.475 |
|  | 2019S | GCA (A-D) | 0.005(0.003) | 0.001(0.000) |  | 0.007(0.004) | 0.012(0.001) | 0.536 |
|  | 2019S | GCA (A-D-E) | 0.001(0.001) | 0.001(0.000) | 0.010(0.007) | 0.005(0.002) | 0.012(0.001) | 0.588 |
|  | 2020S | GCA (A) | 0.005(0.003) |  |  | 0.005(0.001) | 0.017(0.002) | 0.374 |
|  | 2020S | GCA (A-D) | 0.007(0.003) | 0.001(0.000) |  | 0.004(0.001) | 0.013(0.001) | 0.478 |
|  | 2020S | GCA (A-D-E) | 0.002(0.001) | 0.001(0.000) | 0.006(0.003) | 0.003(0.001) | 0.013(0.001) | 0.478 |
| FFFN | 2019S | GCA (A) | 1.205(0.646) |  |  | 1.902(0.791) | 3.828(0.437) | 0.448 |
|  | 2019S | GCA (A-D) | 1.150(0.631) | 0.262(0.153) |  | 1.602(0.717) | 3.415(0.423) | 0.469 |
|  | 2019S | GCA (A-D-E) | 0.297(0.245) | 0.201(0.102) | 1.774(1.308) | 1.477(0.712) | 3.456(0.423) | 0.520 |
|  | 2020S | GCA (A) | 0.490(0.227) |  |  | 0.470(0.134) | 1.900(0.183) | 0.336 |
|  | 2020S | GCA (A-D) | 0.728(0.341) | 0.256(0.125) |  | 0.367(0.117) | 1.317(0.160) | 0.506 |
|  | 2020S | GCA (A-D-E) | 0.172(0.116) | 0.344(0.137) | 0.621(0.289) | 0.336(0.111) | 1.246(0.152) | 0.542 |

As for GCA model, (A) is additive model, (A-D) is additive-dominance model, and (A-D-E) is additive-dominance-epistasis (additive-by-additive) model.

$\sigma_{A}^{2}$,$\sigma_{D}^{2}$ $\sigma_{AA}^{2}$, and$\sigma_{r}^{2}$ are additive, dominance, additive-by-additive variance, and residual genetic components respectively. $\sigma_{\varepsilon}^{2}$ is residual variance.

The estimated posterior of genetic variance components is expressed as mean(standard deviation).
