# Supplementary material for: Genomic Prediction and the Practical Breeding of 12 Quantitative-Inherited Traits in Cucumber (Cucumis sativus L.)
Source: Front Plant Sci. 2021 Aug 24;12:729328. doi: 10.3389/fpls.2021.729328 (PMC8421847; doi:10.3389/fpls.2021.729328)
Supplement: Supplementary file 1 [file Data_Sheet_1.zip › Supplementary Table 5.DOCX]

Supplementary Table 5. The estimated posterior of genetic variance components, broad-sense heritability (*H^2^*) under three GCA models (**without residual genetic component**) for all 12 traits in three seasons

| Season | Trait | Model | Variance components | | | | *H^2^* |
| --- | --- | --- | --- | --- | --- | --- | --- |
|  |  |  | $\sigma_{A}^{2}$ | $\sigma_{D}^{2}$ | $\sigma_{AA}^{2}$ | $\sigma_{\varepsilon}^{2}$ |  |
| cFY | 2018A | GCA (A) | 0.174(0.067) |  |  | 0.313(0.035) | 0.357 |
|  | 2018A | GCA (A-D) | 0.163(0.068) | 0.014(0.008) |  | 0.288(0.036) | 0.382 |
|  | 2018A | GCA (A-D-E) | 0.044(0.051) | 0.011(0.006) | 0.194(0.129) | 0.287(0.034) | 0.465 |
|  | 2019S | GCA (A) | 0.420(0.168) |  |  | 0.442(0.053) | 0.487 |
|  | 2019S | GCA (A-D) | 0.421(0.133) | 0.032(0.024) |  | 0.371(0.046) | 0.55 |
|  | 2019S | GCA (A-D-E) | 0.200(0.201) | 0.021(0.012) | 0.328(0.232) | 0.374(0.044) | 0.595 |
|  | 2020S | GCA (A) | 0.651(0.212) |  |  | 0.449(0.048) | 0.592 |
|  | 2020S | GCA (A-D) | 1.113(0.300) | 0.015(0.007) |  | 0.337(0.036) | 0.770 |
|  | 2020S | GCA (A-D-E) | 0.031(0.026) | 0.012(0.005) | 0.815(0.314) | 0.358(0.039) | 0.706 |
| cFN | 2018A | GCA (A) | 10.577(4.129) |  |  | 20.681(2.412) | 0.338 |
|  | 2018A | GCA (A-D) | 11.084(4.452) | 1.116(0.662) |  | 18.925(2.399) | 0.392 |
|  | 2018A | GCA (A-D-E) | 1.026(0.753) | 0.765(0.435) | 14.804(7.552) | 19.219(2.404) | 0.463 |
|  | 2019S | GCA (A) | 31.838(11.691) |  |  | 34.870(4.076) | 0.477 |
|  | 2019S | GCA (A-D) | 32.609(13.694) | 2.695(1.431) |  | 31.103(3.998) | 0.532 |
|  | 2019S | GCA (A-D-E) | 4.804(5.148) | 2.097(1.463) | 32.596(16.265) | 30.846(3.838) | 0.561 |
|  | 2020S | GCA (A) | 94.456(22.871) |  |  | 48.749(4.634) | 0.660 |
|  | 2020S | GCA (A-D) | 99.856(24.064) | 3.221(1.306) |  | 33.221(3.493) | 0.756 |
|  | 2020S | GCA (A-D-E) | 31.797(33.691) | 2.345(1.052) | 68.336(44.630) | 33.785(3.705) | 0.752 |
| FFT | 2018A | GCA (A) | 22.897(10.258) |  |  | 15.996(1.867) | 0.589 |
|  | 2018A | GCA (A-D) | 22.530(7.954) | 1.211(0.617) |  | 14.148(1.718) | 0.627 |
|  | 2018A | GCA (A-D-E) | 14.153(7.100) | 0.809(0.428) | 8.103(6.229) | 14.444(1.772) | 0.615 |
|  | 2019S | GCA (A) | 8.724(3.344) |  |  | 16.694(2.010) | 0.343 |
|  | 2019S | GCA (A-D) | 9.690(3.369) | 1.277(0.684) |  | 12.399(1.638) | 0.469 |
|  | 2019S | GCA (A-D-E) | 3.090(3.341) | 1.167(0.716) | 11.100(6.654) | 12.398(1.601) | 0.553 |
|  | 2020S | GCA (A) | 2.988(1.022) |  |  | 6.209(0.609) | 0.325 |
|  | 2020S | GCA (A-D) | 3.971(1.171) | 1.316(0.540) |  | 3.332(0.436) | 0.613 |
|  | 2020S | GCA (A-D-E) | 1.363(0.865) | 1.164(0.493) | 2.516(1.200) | 3.298(0.412) | 0.605 |
| cFW | 2018A | GCA (A) | 28.447(15.214) |  |  | 121.349(13.945) | 0.190 |
|  | 2018A | GCA (A-D) | 22.962(12.092) | 3.592(1.661) |  | 117.065(13.416) | 0.185 |
|  | 2018A | GCA (A-D-E) | 8.114(7.091) | 2.684(1.321) | 24.604(16.465) | 118.500(13.925) | 0.230 |
|  | 2019S | GCA (A) | 176.080(50.118) |  |  | 84.895(9.627) | 0.675 |
|  | 2019S | GCA (A-D) | 185.273(51.017) | 5.403(2.363) |  | 82.317(9.629) | 0.698 |
|  | 2019S | GCA (A-D-E) | 141.478(98.969) | 4.559(2.258) | 132.348(169.113) | 82.401(10.005) | 0.772 |
|  | 2020S | GCA (A) | 138.098(33.557) |  |  | 93.758(8.816) | 0.596 |
|  | 2020S | GCA (A-D) | 142.647(33.267) | 5.069(2.097) |  | 87.250(8.914) | 0.629 |
|  | 2020S | GCA (A-D-E) | 98.632(41.199) | 3.997(1.831) | 42.995(40.384) | 87.883(8.981) | 0.624 |
| cFL | 2018A | GCA (A) | 3453.553(993.089) |  |  | 710.552(84.233) | 0.829 |
|  | 2018A | GCA (A-D) | 3426.764(1099.129) | 69.397(29.446) |  | 654.232(76.361) | 0.842 |
|  | 2018A | GCA (A-D-E) | 141.972(123.837) | 52.957(23.743) | 6239.888(1805.595) | 663.362(81.859) | 0.907 |
|  | 2019S | GCA (A) | 4103.959(1415.335) |  |  | 468.646(54.664) | 0.898 |
|  | 2019S | GCA (A-D) | 4107.273(1088.587) | 84.376(35.089) |  | 407.776(53.818) | 0.911 |
|  | 2019S | GCA (A-D-E) | 340.384(504.743) | 57.183(24.071) | 7682.822(3070.018) | 415.565(52.408) | 0.951 |
|  | 2020S | GCA (A) | 2241.984(452.254) |  |  | 335.395(33.738) | 0.870 |
|  | 2020S | GCA (A-D) | 2380.717(444.268) | 43.502(15.028) |  | 295.209(31.646) | 0.891 |
|  | 2020S | GCA (A-D-E) | 1936.305(517.886) | 34.144(14.016) | 402.538(348.872) | 297.134(31.471) | 0.889 |
| cFD | 2018A | GCA (A) | 13.255(4.632) |  |  | 5.783(0.696) | 0.696 |
|  | 2018A | GCA (A-D) | 12.898(3.960) | 0.446(0.225) |  | 5.021(0.628) | 0.727 |
|  | 2018A | GCA (A-D-E) | 8.935(4.643) | 0.341(0.174) | 5.501(6.629) | 5.069(0.629) | 0.745 |
|  | 2019S | GCA (A) | 9.144(2.344) |  |  | 2.324(0.267) | 0.797 |
|  | 2019S | GCA (A-D) | 9.115(2.422) | 0.233(0.129) |  | 2.115(0.253) | 0.816 |
|  | 2019S | GCA (A-D-E) | 0.292(0.235) | 0.158(0.072) | 18.920(5.806) | 2.134(0.260) | 0.901 |
|  | 2020S | GCA (A) | 3.413(0.919) |  |  | 1.702(0.169) | 0.667 |
|  | 2020S | GCA (A-D) | 3.387(0.772) | 0.091(0.038) |  | 1.516(0.155) | 0.696 |
|  | 2020S | GCA (A-D-E) | 3.355(0.898) | 0.071(0.035) | 0.591(0.286) | 1.533(0.155) | 0.724 |
| cFNL | 2018A | GCA (A) | 111.326(34.455) |  |  | 36.358(4.239) | 0.754 |
|  | 2018A | GCA (A-D) | 125.138(35.199) | 4.472(2.228) |  | 30.882(4.088) | 0.808 |
|  | 2018A | GCA (A-D-E) | 42.775(42.233) | 3.729(2.393) | 124.163(110.710) | 31.688(4.150) | 0.843 |
|  | 2019S | GCA (A) | 199.930(61.568) |  |  | 46.562(5.351) | 0.811 |
|  | 2019S | GCA (A-D) | 188.329(52.588) | 7.963(3.574) |  | 33.722(4.613) | 0.853 |
|  | 2019S | GCA (A-D-E) | 12.284(11.985) | 6.568(3.293) | 374.670(117.053) | 34.616(4.744) | 0.919 |
|  | 2020S | GCA (A) | 181.838(42.651) |  |  | 44.871(4.559) | 0.802 |
|  | 2020S | GCA (A-D) | 179.057(47.826) | 6.035(2.277) |  | 35.195(3.939) | 0.840 |
|  | 2020S | GCA (A-D-E) | 145.826(36.942) | 4.267(1.867) | 21.995(9.056) | 36.392(4.120) | 0.825 |
| cFTH | 2018A | GCA (A) | 0.023(0.010) |  |  | 0.132(0.015) | 0.147 |
|  | 2018A | GCA (A-D) | 0.023(0.012) | 0.005(0.003) |  | 0.128(0.016) | 0.178 |
|  | 2018A | GCA (A-D-E) | 0.005(0.004) | 0.003(0.002) | 0.028(0.014) | 0.127(0.015) | 0.219 |
|  | 2019S | GCA (A) | 0.224(0.078) |  |  | 0.184(0.021) | 0.549 |
|  | 2019S | GCA (A-D) | 0.264(0.099) | 0.011(0.006) |  | 0.166(0.021) | 0.623 |
|  | 2019S | GCA (A-D-E) | 0.009(0.007) | 0.008(0.005) | 0.429(0.184) | 0.171(0.021) | 0.724 |
|  | 2020S | GCA (A) | 0.172(0.055) |  |  | 0.121(0.012) | 0.586 |
|  | 2020S | GCA (A-D) | 0.202(0.051) | 0.006(0.004) |  | 0.110(0.012) | 0.654 |
|  | 2020S | GCA (A-D-E) | 0.047(0.061) | 0.004(0.003) | 0.146(0.094) | 0.113(0.012) | 0.636 |
| cSCR | 2018A | GCA (A) | 1.903(0.709) |  |  | 0.853(0.101) | 0.691 |
|  | 2018A | GCA (A-D) | 2.208(0.985) | 0.064(0.030) |  | 0.750(0.090) | 0.752 |
|  | 2018A | GCA (A-D-E) | 0.827(0.718) | 0.048(0.025) | 1.585(1.574) | 0.763(0.093) | 0.763 |
|  | 2019S | GCA (A) | 0.542(0.142) |  |  | 0.211(0.024) | 0.720 |
|  | 2019S | GCA (A-D) | 0.530(0.164) | 0.015(0.007) |  | 0.200(0.025) | 0.732 |
|  | 2019S | GCA (A-D-E) | 0.032(0.025) | 0.012(0.007) | 0.995(0.352) | 0.202(0.024) | 0.837 |
|  | 2020S | GCA (A) | 0.259(0.064) |  |  | 0.140(0.013) | 0.65 |
|  | 2020S | GCA (A-D) | 0.284(0.068) | 0.009(0.004) |  | 0.112(0.012) | 0.722 |
|  | 2020S | GCA (A-D-E) | 0.065(0.088) | 0.007(0.004) | 0.262(0.149) | 0.115(0.012) | 0.745 |
| cFSD | 2018A | GCA (A) | 0.406(0.114) |  |  | 0.232(0.027) | 0.637 |
|  | 2018A | GCA (A-D) | 0.433(0.183) | 0.020(0.012) |  | 0.202(0.026) | 0.692 |
|  | 2018A | GCA (A-D-E) | 0.114(0.160) | 0.014(0.007) | 0.584(0.414) | 0.207(0.026) | 0.775 |
|  | 2019S | GCA (A) | 0.225(0.066) |  |  | 0.055(0.006) | 0.802 |
|  | 2019S | GCA (A-D) | 0.186(0.056) | 0.005(0.002) |  | 0.048(0.006) | 0.799 |
|  | 2019S | GCA (A-D-E) | 0.197(0.058) | 0.004(0.002) | 0.030(0.022) | 0.049(0.006) | 0.826 |
|  | 2020S | GCA (A) | 0.205(0.054) |  |  | 0.127(0.012) | 0.617 |
|  | 2020S | GCA (A-D) | 0.200(0.047) | 0.009(0.005) |  | 0.114(0.012) | 0.647 |
|  | 2020S | GCA (A-D-E) | 0.185(0.057) | 0.008(0.006) | 0.035(0.019) | 0.114(0.013) | 0.665 |
| FFNR | 2019S | GCA (A) | 0.015(0.007) |  |  | 0.016(0.002) | 0.494 |
|  | 2019S | GCA (A-D) | 0.014(0.006) | 0.001(0.000) |  | 0.012(0.001) | 0.568 |
|  | 2019S | GCA (A-D-E) | 0.005(0.003) | 0.001(0.000) | 0.011(0.006) | 0.012(0.001) | 0.579 |
|  | 2020S | GCA (A) | 0.011(0.004) |  |  | 0.017(0.002) | 0.386 |
|  | 2020S | GCA (A-D) | 0.011(0.003) | 0.001(0.001) |  | 0.013(0.001) | 0.493 |
|  | 2020S | GCA (A-D-E) | 0.004(0.003) | 0.001(0.000) | 0.008(0.004) | 0.013(0.001) | 0.510 |
| FFFN | 2019S | GCA (A) | 4.149(1.475) |  |  | 3.939(0.455) | 0.513 |
|  | 2019S | GCA (A-D) | 5.239(2.322) | 0.329(0.175) |  | 3.446(0.442) | 0.618 |
|  | 2019S | GCA (A-D-E) | 0.477(0.428) | 0.229(0.110) | 6.846(3.229) | 3.515(0.424) | 0.682 |
|  | 2020S | GCA (A) | 1.501(0.415) |  |  | 1.922(0.189) | 0.438 |
|  | 2020S | GCA (A-D) | 1.783(0.499) | 0.354(0.171) |  | 1.258(0.157) | 0.630 |
|  | 2020S | GCA (A-D-E) | 0.664(0.486) | 0.329(0.150) | 1.053(0.592) | 1.274(0.158) | 0.616 |

As for GCA model, (A) is additive model, (A-D) is additive-dominance model, and (A-D-E) is additive-dominance-epistasis (additive-by-additive) model.

$\sigma_{A}^{2}$,$\sigma_{D}^{2}$ and $\sigma_{AA}^{2}$ are additive, dominance, additive-by-additive variance component respectively. $\sigma_{\varepsilon}^{2}$ is residual variance.

The estimated posterior of genetic variance components is expressed as mean(standard deviation).
