# Supplementary figures and images for: Genomic Prediction and the Practical Breeding of 12 Quantitative-Inherited Traits in Cucumber (Cucumis sativus L.)
Source: Front Plant Sci. 2021 Aug 24;12:729328. doi: 10.3389/fpls.2021.729328 (PMC8421847; doi:10.3389/fpls.2021.729328)

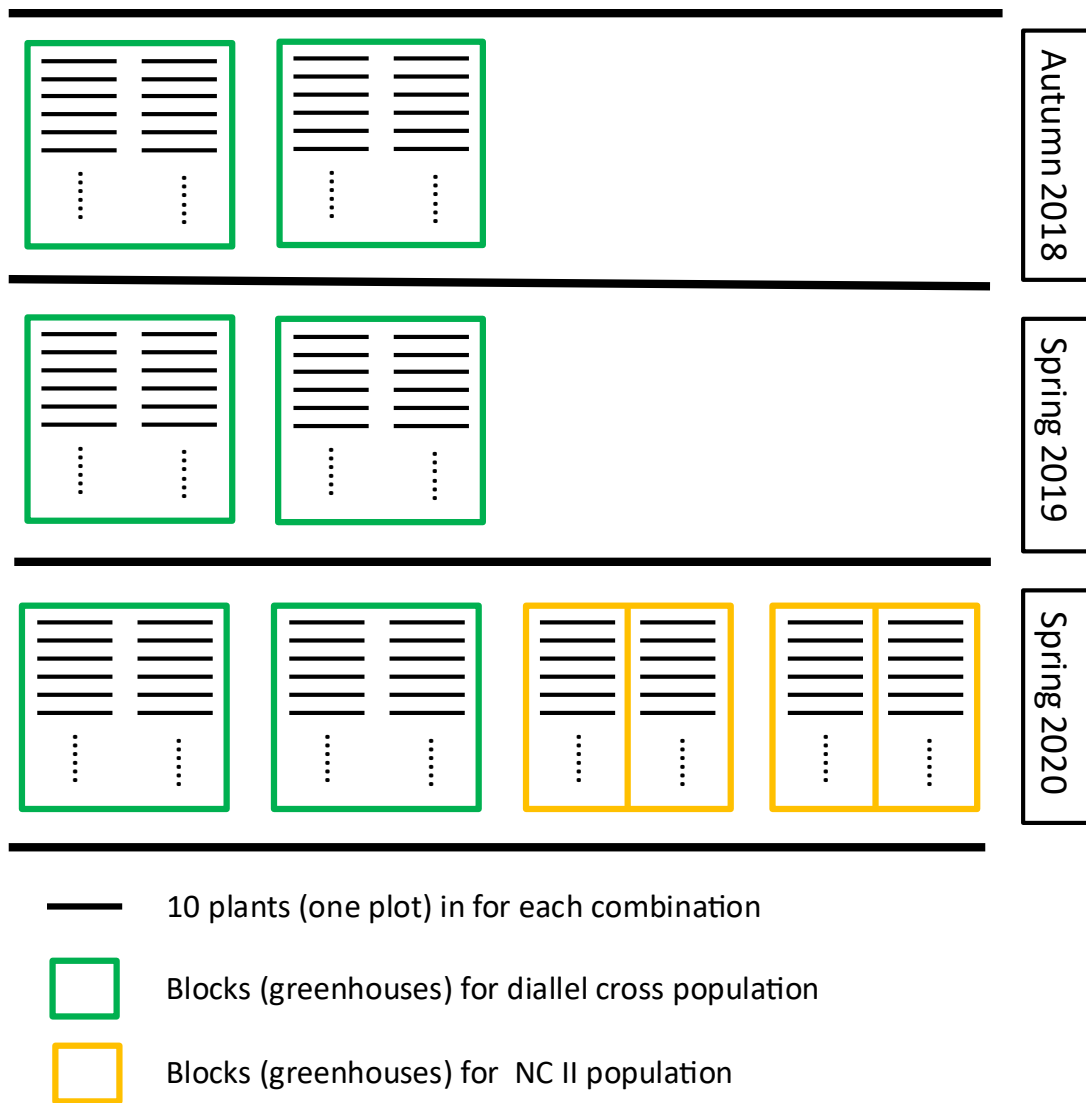

**Supplementary Figure 1.** The field experiment design of this study

Supplement: Supplementary file 1 [file Data_Sheet_1.zip › Supplementary Figure 1.PDF]
